# Expression and Functional Analysis of the Compact Thermophilic *Anoxybacillus flavithermus* Cas9 Nuclease

**DOI:** 10.3390/ijms242317121

**Published:** 2023-12-04

**Authors:** Anastasiya Matveeva, Alexander Ryabchenko, Viktoria Petrova, Daria Prokhorova, Evgenii Zhuravlev, Alexander Zakabunin, Artem Tikunov, Grigory Stepanov

**Affiliations:** Institute of Chemical Biology and Fundamental Medicine, Siberian Branch of the Russian Academy of Sciences, Novosibirsk 630090, Russia; anastasiya.maatveeva@gmail.com (A.M.); petrova.v.a@biolabmix.ru (V.P.); evgenijur@gmail.com (E.Z.); arttik1986@gmail.com (A.T.)

**Keywords:** CRISPR/Cas9, genome engineering, whole-genome sequencing, PAM identification

## Abstract

Research on Cas9 nucleases from different organisms holds great promise for advancing genome engineering and gene therapy tools, as it could provide novel structural insights into CRISPR editing mechanisms, expanding its application area in biology and medicine. The subclass of thermophilic Cas9 nucleases is actively expanding due to the advances in genome sequencing allowing for the meticulous examination of various microorganisms’ genomes in search of the novel CRISPR systems. The most prominent thermophilic Cas9 effectors known to date are GeoCas9, ThermoCas9, IgnaviCas9, AceCas9, and others. These nucleases are characterized by a varying temperature range of the activity and stringent PAM preferences; thus, further diversification of the naturally occurring thermophilic Cas9 subclass presents an intriguing task. This study focuses on generating a construct to express a compact Cas9 nuclease (AnoCas9) from the thermophilic microorganism *Anoxybacillus flavithermus* displaying the nuclease activity in the 37–60 °C range and the PAM preference of 5′-NNNNCDAA-3′ in vitro. Here, we highlight the close relation of AnoCas9 to the GeoCas9 family of compact thermophilic Cas9 effectors. AnoCas9, beyond broadening the repertoire of Cas9 nucleases, suggests application in areas requiring the presence of thermostable CRISPR/Cas systems in vitro, such as sequencing libraries’ enrichment, allele-specific isothermal PCR, and others.

## 1. Introduction

Clustered Regularly Interspaced Short Palindromic Repeats (CRISPR) systems provide microbes with an RNA-based mechanism for innate adaptive immunity against foreign genetic elements. Typically, their genomic context comprises the CRISPR locus containing spacer sequences interspersed with repeats, and arrays of CRISPR-associated (*cas*) genes encoding functional proteins. In the presence of foreign DNA, CRISPR/Cas integrates its short fragment into the CRISPR locus as a new spacer. Expression of the CRISPR locus with further processing leads to the formation of mature CRISPR RNAs (crRNAs). Cas proteins expressed from the *cas* array then bind to crRNA to form ribonucleoprotein complexes (RNPs). crRNA specifically targets invading nucleic acid and guides RNP in order to disrupt it [1,2,3]. Class 2 systems are characterized by the presence of a single RNA-bound Cas effector that recognizes and cleaves target DNA [4,5]. Their programmable nature has been employed to develop novel strategies for specific genome editing using CRISPR/Cas9 (class 2 type II), CRISPR/Cas12 (class 2 type V), and, later, CRISPR/Cas13 (class 2 type VI) systems [5,6,7,8,9,10,11,12]. The expansion of versatile tools and approaches based on CRISPR/Cas gave rise to the robust evolution of genome engineering with applications in medicine, biotechnology, diagnostics, and fundamental research [13,14,15,16,17,18,19,20,21,22,23,24].

A key component of the class 2 type II CRISPR/Cas systems is a single multidomain enzyme Cas9. It functions using either the duplex of guide crRNA and trans-activating crRNA (tracrRNA) or the product of their artificial fusion in the form of a single-guide RNA (sgRNA) [5,25]. The Cas9 RNP complex utilizes two nuclease sites, RuvC and HNH, to generate a double-strand break (DSB) in a target DNA sequence. DSB sites are determined through the complementarity of the target DNA to the 20-nt protospacer sequence in guide RNA (crRNA or sgRNA). The re-programming of Cas9 to aim the cleavage of the DNA of choice, however, is limited by the requirement of a specific protospacer adjacent motif (PAM) sequence. The presence of PAM in the target DNA region is necessary both for the recognition and the initiation of cleavage. Each Cas9 effector associates with a specific PAM sequence, making it a unique signature [4,5]. The most well-known and extensively used for the programmed genome editing CRISPR/Cas9 system is derived from *Streptococcus pyogenes* (SpyCas9). It has been adapted to suit multiple applications requiring the introduction of DSBs or single-strand breaks (nicks) into target DNA, or its binding [6,26,27,28,29,30]. SpyCas9 prefers a 5′-NGG-3′ sequence as its PAM motif, thus setting restrictions for the range of possible DNA targets. Therefore, there is a need to expand the Cas9 toolkit with novel proteins exhibiting diverse PAM specificities. Moreover, the discovery and characterization of other naturally occurring CRISPR/Cas9 systems are quite promising in terms of obtaining Cas effectors with unique recognition and cleavage properties that would allow for the conditional regulation of the editing process and expand the application of CRISPR in multiple areas, including fundamental structural studies [31,32].

Routine analyses of large sets of sequencing data revealed that multiple prokaryotic genomes incorporate CRISPR arrays, including CRISPR/Cas9 [31,33]. Interestingly, almost 50% of the described Cas9 proteins fall into the type II-C category. This type has been presumed to display a lesser nuclease activity in comparison to the type II-A proteins, with SpyCas9 being the most prominent exemplar. Nevertheless, recent studies demonstrate a larger diversity in both the structure and properties of the type II-C proteins. This subset of Cas9 proteins is characterized by a smaller size, which is advantageous for their delivery into cells using vector-based approaches, and enhanced specificity due to the elongated PAM sequence or, in some cases, due to the greater length of the crRNA:target DNA heteroduplex [34]. The majority of organisms that have been assigned as type II-C CRISPR hosts are typically classified as extremophiles inhabiting hot springs, wastewaters, etc. The most well-known representative of this type is Cas9 derived from *Geobacillus stearothermophilus*, which has demonstrated editing activity in mammalian cells, a wide range of working temperatures, and increased thermostability alongside an enhanced lifetime in human plasma [34,35].

*Anoxybacillus* species are frequently discovered in thermophilic conditions. As an example, *Anoxybacillus flavithermus* inhabits hot springs and is characterized as a Gram-positive, endospore-forming anaerobic microorganism [36]. To date, there are only seven whole genomes for *Anoxybacillus flavithermus* strains deposited in genome databases. Furthermore, sequence analysis revealed the presence of a common gene group playing a role in the adaptation to environmental conditions, such as growth in the alkaline environment under high temperatures. The search for CRISPR/Cas systems resulted in the discovery of genes associated with class 1 (types I and III) and class 2 (type V) for some of the strains; however, corresponding arrays have not been yet studied extensively to assess the functioning of CRISPR immunity in these microorganisms [37]. Thus, further investigations into thermophilic microorganisms hold great promise for expanding the CRISPR toolbox.

To identify putative functioning CRISPR systems, the present study analyzed the shotgun whole-genome sequencing data for the set of microorganisms deposited in the Collection of Extremophilic Microorganisms and Type Cultures (Institute of Chemical Biology and Fundamental Medicine, Novosibirsk). The *Anoxybacillus flavithermus* strain obtained in the Kamchatka region was selected since its genome contains the Cas9 ORF sequence as well as the adjacent CRISPR array. Further optimization of the protein-coding sequence enabled the successful expression of the *Anoxybacillus flavithermus* Cas9 (AnoCas9, “Kamchatskii”) in *E. coli*. AnoCas9 demonstrated stability and nuclease activity at high temperatures in vitro. The CRISPR array was analyzed to predict the PAM sequence, and the prediction was further supported with in vitro experiments based on the cleavage of fluorescently labeled dsDNA substrates. The 5′-NNNNCDAA-3′ sequence was identified as the functional PAM motif. This paper is easy-to-use for researchers interested in the specific details regarding the cloning, expression, the characterization of novel Cas effectors since Section 4 gives a meticulous description of the approaches used. On the other hand, Section 2 reveals the unique modifications of the protocol for the successful expression of AnoCas9. Researchers might find great use of the provided method for the PAM determination since it does not require the NGS techniques. Finally, the Section 3 highlights the characterization of yet another thermophilic and thermostable Cas9, its promising areas of applications, and possible future investigations including mechanistic ones.

## 2. Results

### 2.1. Identification of Anoxybacillus flavithermus Cas9

Twenty-one bacteria strains were selected from the Collection of Extremophilic Microorganisms and Type Cultures (Institute of Chemical Biology and Fundamental Medicine, Novosibirsk, Russia). The strains were cultivated, and genomic DNA was isolated for further sequencing. After the analysis of RAST annotations, eight samples were found to contain sequences encoding various Cas proteins with only seven out of them containing full-sized CRISPR-Cas arrays consisting of *cas* genes and spacer-repeat units. Further analysis informed the selection of the *Anoxybacillus flavithermus* strain 4025, as the Cas9 ORF (further referred to as *anoCas9*) was found and annotated in its genome sequencing data.

**Figure 1:**
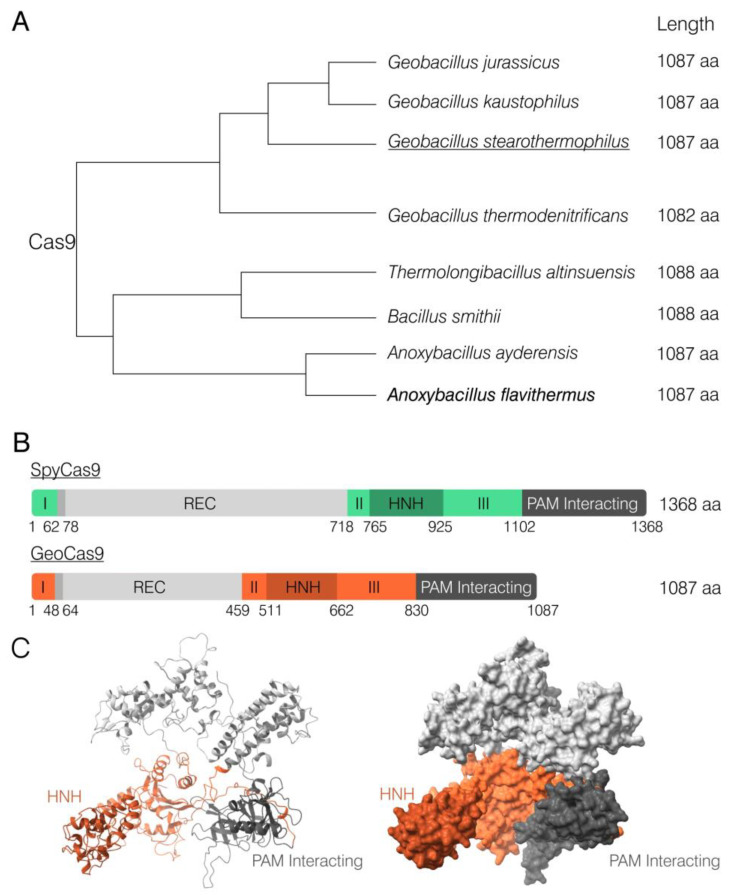
Schematical representation of the AnoCas9 BLASTp alignment analysis (**A**). Comparison of SpyCas9 and GeoCas9 domain structures as previously described [35] (**B**). Protein model of AnoCas9 built with the Phyre2 tool and visualized in ChimeraX [38,39] (**C**).

The *Anoxybacillus flavithermus* Cas9 ORF is 3264-nt long and encodes a protein of 1087 aa in length. BLASTn analysis using the whole-genome shotgun contigs collection revealed a single fully matching nucleotide sequence that had already been annotated as a Cas9 nuclease. It was found through a similar bioinformatical analysis of the genome of another *Anoxybacillus flavithermus* strain (GenBank WP_099668921.1). BLASTp analysis of AnoCas9 demonstrated its high homology with a few other *Anoxybacillus* Cas9, as well as with other species (81.86–97.33%) (Figure S1). Interestingly, all of these proteins were characterized by a smaller size than commonly used SpyCas9 (Figure 1A). AnoCas9 was also of the same length as *Geobacillus stearothermophilus* Cas9 (GeoCas9), one of the most prominent and well-described thermostable Cas effectors [35]. The domain structure of GeoCas9 was established previously, with the REC domain being considerably smaller compared to SpyCas9 (Figure 1B,C and Figure S1). Although high sequence homology between AnoCas9 and GeoCas9 suggested similar properties, in vitro experiments were further required to properly characterize the AnoCas9 effector.

### 2.2. Obtaining AnoCas9 Recombinant Protein

#### 2.2.1. Cloning *anoCas9* Gene into pETm Vector

The *anoCas9* ORF was first cloned into a pETm vector—a variant of pET36b(+) in which the original ORF between start- and stop-codons is replaced with the modified ORF that contains restriction sites and DNA fragment encoding eight histidine amino acid residues required for further IMAC purification procedures [40]. The *anoCas9* gene was amplified from the *Anoxybacillus flavithermus* genomic DNA using a set of primers overlapping with pETm, pre-cleaved by HindIII. Routine Gibson assembly was then carried out, followed by the transformation of electrocompetent *E. coli* cells. The colonies containing pETm with *anoCas9* insertion (pETm-AnoCas9) were selected and used to inoculate LB media for overnight incubation and subsequent plasmid DNA isolation. Plasmid DNA was then Sanger-sequenced to verify the insertion sequence (Figure S2).

*anoCas9* codons were analyzed using the ATGme tool for the expression in *E. coli* cells. Its ORF was found to contain a lot of rare *E. coli* codons, and, thus, Rosetta 2 (DE3) was selected for the expression as a more prolific strain. Sanger-verified pETm-AnoCas9 vector was used to transform Rosetta 2 (DE3) cells. Colonies were screened and tested for protein expression, with the expected molecular weight of AnoCas9-8xHis being ~130.5 kDa. However, for 20 clones, no significant difference was observed between samples before and after the induction with IPTG, and there was no band corresponding to the expected recombinant AnoCas9. We suggest that the lack of expression might be caused by the presence of rare *E. coli* codons in the wild-type *anoCas9* ORF.

#### 2.2.2. 5′-End *anoCas9* Codon Optimization

**Figure 2:**
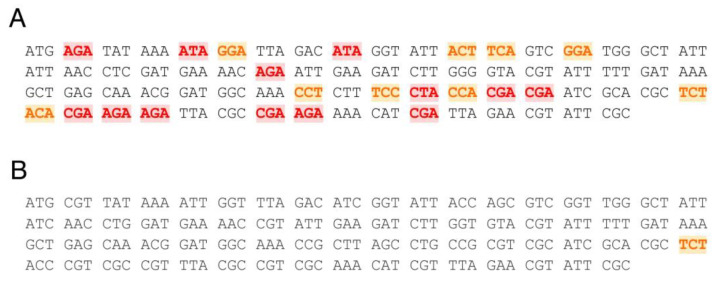
The structure of the first 70 *anoCas9* codons before (**A**) and after the optimization (**B**). Rare codons (frequency from 5 to 10) are highlighted in orange and very rare codons (frequency less than 5) are in red.

Sequence analysis demonstrated the presence of multiple rare *E. coli* codons in the 5′-end region of *anoCas9* ORF. Moreover, rare codons were found in repeats, potentially causing premature termination of protein elongation. To increase the recombinant AnoCas9 expression efficiency in *E. coli*, the first 70 codons were optimized using the ATGme tool (Figure 2).

The optimized DNA fragment was synthesized de novo and used to assemble a full-length optimized *anoCas9*. The *anoCas9* gene was also fused with the maltose-binding protein (MBP) gene to increase the efficiency of chimeric recombinant polypeptide MBP-AnoCas9 synthesis.

#### 2.2.3. Cloning Partially Optimized *anoCas9* Gene into pD441-HMBP and Producing Recombinant AnoCas9

To generate the construct encoding the fused *MBP-anoCas9* gene, the pD441-HMBP vector was selected. The vector contained a DNA fragment encoding 6xHis at the 5′-end of the MBP gene (HMBP-tag) and a DNA fragment downstream of the MBP gene encoding TEVp protease that enabled the cleavage of the purified protein in order to obtain HMBP and AnoCas9.

Routine Gibson assembly was carried out using three fragments: two amplicons and a digested pD441-HMBP vector (Section 4.3 Materials and Methods). A ligation mixture was then used to transform electrocompetent TOP10 *E. coli* cells. PCR screening with the flanking primers allowed us to select colonies carrying the pD441-HMBP-AnoCas9 vector (Figure S3). Clones of choice were further cultivated to isolate plasmid DNA followed by Sanger sequencing to verify the structure of the DNA fragment encoding the optimized *anoCas9* gene (Figure S4).

The generated pD441-HMBP-AnoCas9 vector was used to transform Rosetta 2 (DE3) cells. Clones were selected with PCR screening, and their ability to produce the protein was assessed. The molecular weight (Mw) of the recombinant chimeric HMBP-AnoCas9 polypeptide was expected to be ~173 kDa. IPTG induction resulted in the formation of a band with mobility corresponding to the expected Mw, thus indicating the presence of the target HMBP-AnoCas9 polypeptide.

**Figure 3:**
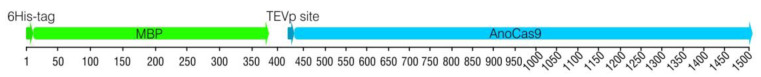
Schematic representation of the HMBP-AnoCas9 structure.

Selected clones were then used to grow the biomass for the recombinant protein production, as described in Section 4.4, and to purify the obtained recombinant protein, as described in Section 4.5 (Materials and Methods). After the first purification stage using the IMAC Sepharose, a fused HMBP-AnoCas9 polypeptide was obtained (Figure 3).

**Figure 4:**
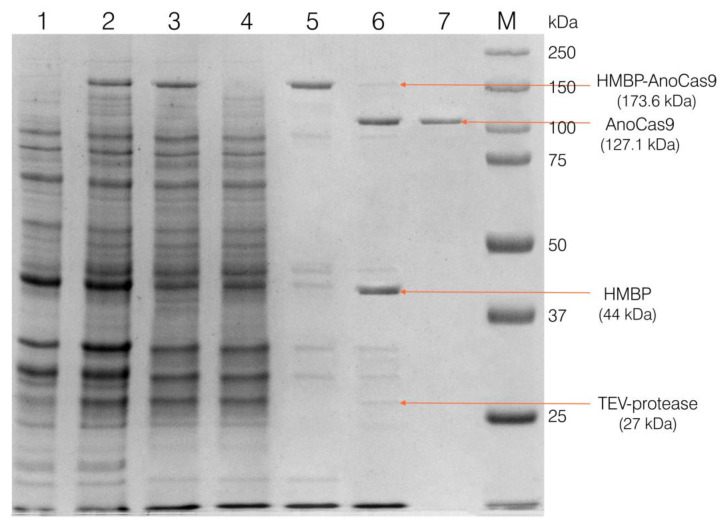
Analysis of the fractions at different purification stages in 10% SDS-PAGE. Lane 1 refers to cells with no IPTG induction; lane 2—cells induced with IPTG (biomass for the protein isolation); lane 3—cell extract (supernatant after sonication and centrifugation); lane 4—the unretained fraction after IMAC chromatography; lane 5—purified HMBP-AnoCas9 after the IMAC; lane 6—reaction mixture after incubation with TEV protease; lane 7—purified AnoCas9 after SP Sepharose chromatography (final solution).

The polypeptide was then cleaved with TEV protease to separate the HMBP-tag and AnoCas9 protein, with the latter being further purified using SP Sepharose chromatography and concentrated as described in Section 4.5.3 to produce the final solution (Figure 4).

### 2.3. CRISPR Array Analysis

To assess the biological activity of the produced AnoCas9 protein, its guide RNA structure had to be determined as well as a functional PAM sequence crucial for Cas9 nuclease activity. Whole-genome sequencing data for the *Anoxybacillus flavithermus* strain of choice were analyzed using the RAST and CRISPR-Finder tools to identify the CRISPR array associated with AnoCas9. The array consisted of 16 spacers and repeats (Table S2).

**Figure 5:**
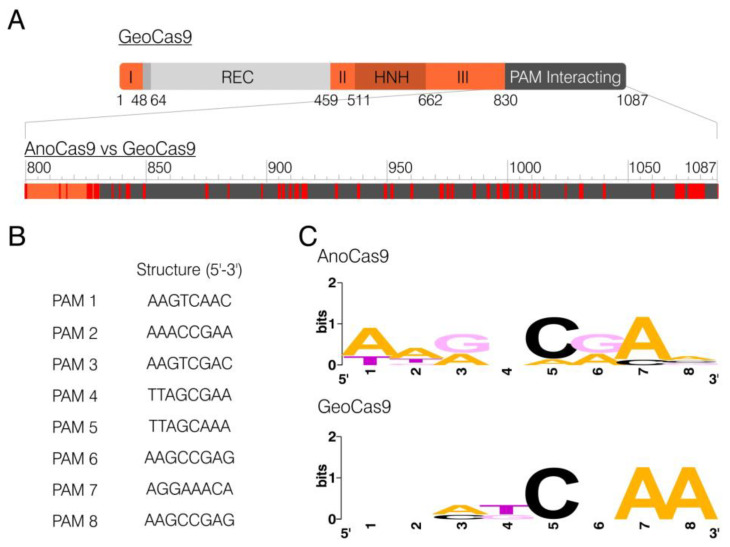
PAM motif prediction. BLASTp alignment between AnoCas9 and GeoCas9; mismatches are highlighted in red (**A**). PAM sequences derived from target sequences generated with CRISPRTarget (**B**). Weblogo analysis of PAM sets for AnoCas9 and GeoCas9 (**C**).

The domain structure of GeoCas9 has been previously established, with the PAM-interacting (PI) domain being located in the C-terminal region (831–1087 aa) [35]. The alignment of AnoCas9 and GeoCas9 peptide sequences revealed 52 mismatches in the PI domain, 25 of which were non-conservative (Figure 5A). This discovery led to the assumption that AnoCas9 might require a PAM motif different from the GeoCas9 motif (5′-NNNNCRAA-3′, where N is any nucleotide). While multiple approaches are used nowadays to identify the PAM, most of them rely on in vitro experiments, often including the usage of libraries and a subsequent NGS analysis. The CRISPRTarget tool, on the other hand, helps predict the PAM through the bioinformatical analysis of spacer sequences. The analysis was carried out using the standard parameters to align spacer sequences with the data from Genbank-Phage, RefSeq-plasmid, and IMGVR databases. The 20 most reliable alignments were generated, and all of them used data from IMGVR. Eight targets with >90% match were selected; however, they referred to two spacers only—spacer 3 and spacer 13 (Figure S5). The analysis of the 3′-adjacent region resulted in the selection of putative PAM sequences (Figure 5B). A subsequent analysis using WebLogo allowed us to predict the overall PAM motif—5′-NNRNCRAN-3′. Interestingly, this sequence aligned with the GeoCas9 motif 5′-NNNNCNAA-3′ (Figure 5C).

**Figure 6:**
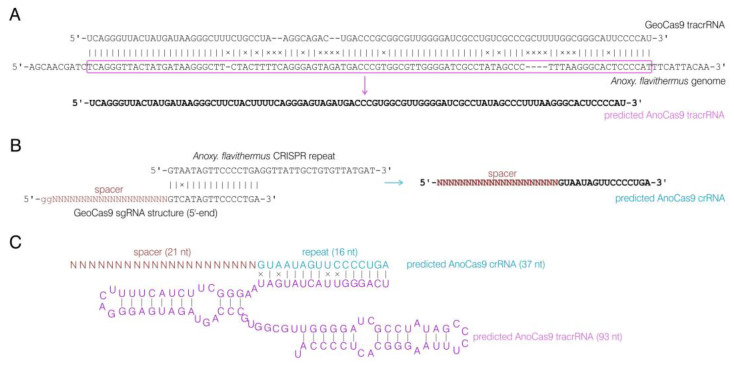
Analysis of the AnoCas9 CRISPR array. GeoCas9 tracrRNA and *A. flavithermus* whole-genome shotgun sequencing alignment and predicted AnoCas9 tracrRNA structure (**A**). *A. flavithermus* CRISPR repeat alignment with the GeoCas9 sgRNA and predicted AnoCas9 crRNA structure (**B**). Overall structure of the functional AnoCas9 crRNA:tracrRNA duplex (**C**).

Given the high similarity observed between AnoCas9 and GeoCas9, we hypothesized that the guide RNA structure might also bear a resemblance. The sequence of tracrRNA for the GeoCas9 nuclease found previously [35] was aligned to the whole-genome sequencing data for the *Anoxybacillus flavithermus* strain using BLASTn. As a result, a homologous sequence with 78% similarity was observed in the CRISPR array vicinity and was further used as the AnoCas9 tracrRNA sequence (Figure 6A). This sequence (93 nt) was found to be shorter than GeoCas9 tracrRNA (98 nt). By analyzing tracrRNA, spacer sequences, and the GeoCas9 sgRNA structure described in previously published data, we predicted the structure of crRNA for AnoCas9 with the 21-nt guide region (Figure 6B). While experiments involving the high-throughput sequencing of small RNA fraction could be carried out in the future to support this prediction, for the purposes of the current study, the predicted crRNA and tracrRNA structures (Figure 6C) were synthesized and used to assess AnoCas9 properties in vitro.

### 2.4. Recombinant AnoCas9 Nuclease Activity Assessment

**Figure 7:**
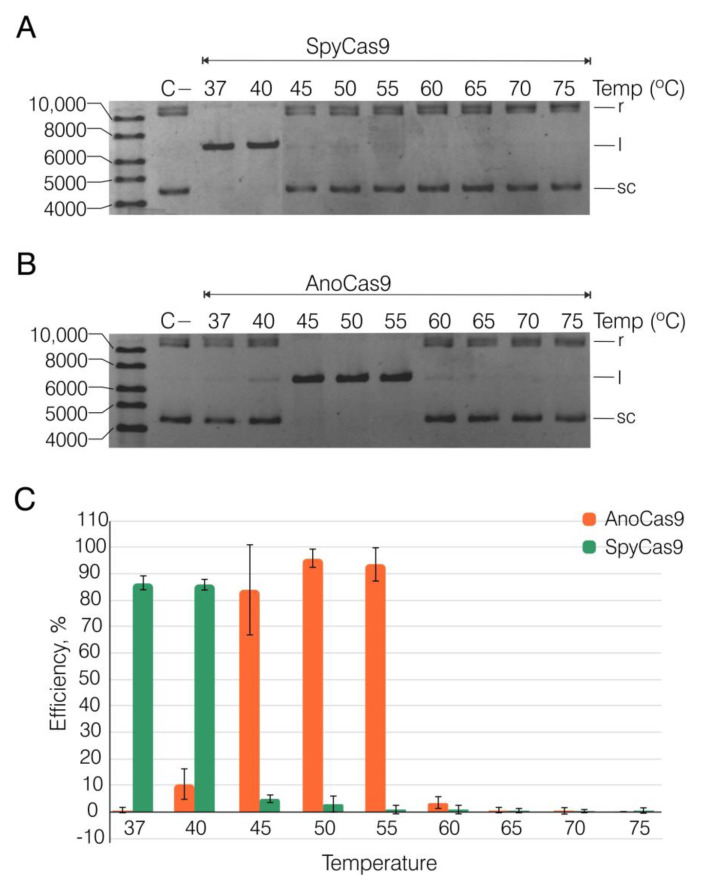
Assessment of SpyCas9 (**A**) and AnoCas9 (**B**) activity. Reaction mixtures were resolved in the 1% agarose gel after incubation at the indicated temperature and quenching. Plasmid DNA without Cas9 treatment (C−) was used as a negative control. The linear [l] band represents the cleavage product, while the top and bottom bands represent the substrate (relaxed [r] and supercoiled [sc] forms). The molar ratio of Cas9 RNP to target plasmid was 50:1, and the cleavage reactions were stopped after 60 min. Three replicates were used to assess the editing efficacy in the experimental temperature range (**C**). The efficiency was calculated as mentioned (p. 4.8, Materials and methods). Data are presented as mean ± standard deviation (SD).

To assess the cleavage activity of AnoCas9, we used SpyCas9 as the control nuclease and the plasmid substrate pANXA6 from our previous study [41]. The 5′-TGTTCAAA-3′ PAM motif fitting the described GeoCas9 motif (5′-NNNNCRAA-3′) was selected to construct crRNA. This design implied that sequence and structural homology between AnoCas9 and GeoCas9 would result in similar activity. We tested the efficiency of SpyCas9 and AnoCas9 RNPs within a wide temperature range (37–75 °C, Figure 7 and Figure S6). The reaction was carried out on the target plasmid in the presence of a 50-fold excess of Cas9/crRNA:tracrRNA and stopped after 60 min. SpyCas9 was most active at 37 and 40 °C, and the percentage of cleaved plasmid reached up to 86% (Figure 7A,C). At the same time, an increase in temperature led to a marked slowdown of the plasmid cleavage, which is consistent with previous data [35]. In contrast, AnoCas9 retained its nuclease activity at temperatures up to 60 °C (Figure 7B,C). Interestingly, the percentage of cleaved plasmid notably increased at temperatures in the 45–55 °C range, reaching its maximum of 96% at 50 °C (Figure 7C), with AnoCas9 activity reduced to a low level at temperatures above 65 °C. Thus, AnoCas9 represents another thermostable Cas9 ortholog.

### 2.5. In Vitro PAM Analysis

While we successfully demonstrated the nuclease activity of AnoCas9 under mesophilic conditions, the question of PAM preferences was still left for discussion. In order to support the CRISPRTarget findings, we constructed an array of FAM-labeled dsDNA templates with the spacer sequence used in the previous step as a target in the pANXA6 plasmid. The PAM motif 5′-TGTTCAAA-3′, present in pANXA6 and used for AnoCas9-mediated cleavage, was chosen as a template sequence. Given that CRISPRTarget analysis generated 5′-NNRNCRAN-3′ as the general structure for the AnoCas9 CRISPR array, cytidine in position 5 and adenosine in position 7 were further locked, and other positions were randomized in pairs (Table S3).

**Figure 8:**
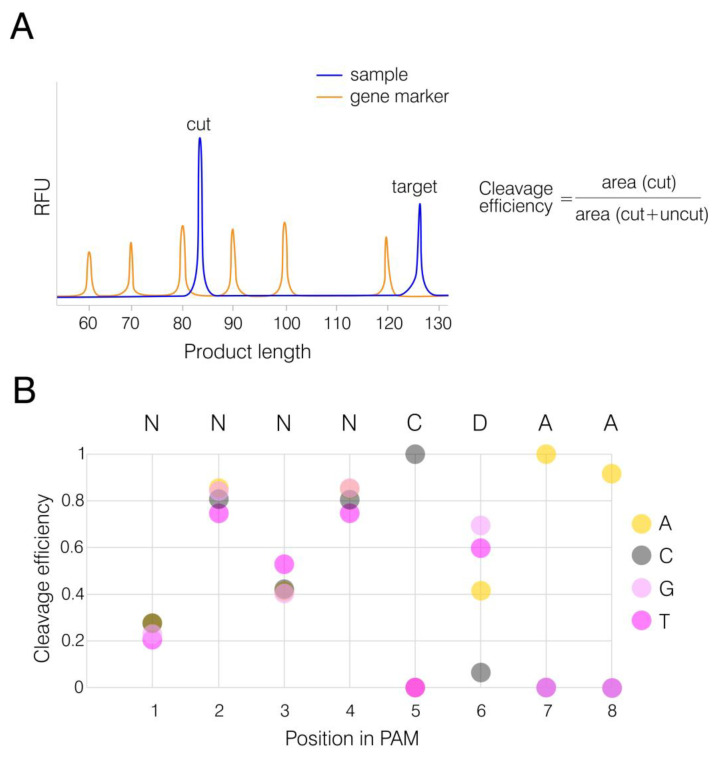
PAM determination. Electropherogram demonstrating the cleavage of target dsDNA substrate containing a specific PAM sequence (**A**). FAM-labeled dsDNA (“target”) was digested by Cas9. The presence of a functional PAM in the substrate structure resulted in the formation of short fragments (“cut”), with areas of peaks referring to the “cut” and “uncut” products measured to assess the cleavage efficiency. Analysis of PAM preferences for AnoCas9 based on the previously established TGTTCAAA sequence and the CRISPRTarget prediction (**B**). Substrates with variable nucleotides in different positions were tested. The average cleavage efficiency was assessed for each nucleotide position in pairs, except for locked positions 5 and 7.

Templates were synthesized through two consequent heteroduplex elongation and amplification reactions as described in Section 4.9 (Materials and Methods). AnoCas9 RNP complexes were pre-formed and incubated with dsDNA substrates at 50 °C for 1 h, as it was considered an optimal temperature. The reaction was then stopped and resolved using a gene analyzer. The uncleaved substrate length was expected to be 126 bp, while the AnoCas9 cleavage led to the formation of a FAM-labeled product with the expected length of 84 bp (Figure 8A and Figure S7). The area ratio, cut to total (cut+uncut), was calculated to assess the AnoCas9 editing efficiency (Figure 8B). The analysis revealed a PAM preference for 5′-NNNNCDAA-3′, thus supporting the bioinformatical prediction described above and indicating that AnoCas9 belongs to the GeoCas9 subclass of thermophilic Cas9 nucleases.

## 3. Discussion

Currently, researchers are still exploring a pool of thermostable Cas9 nucleases, with novel proteins being discovered and characterized. However, this subclass of Cas effectors has already found its application in editing genomes of thermophilic microorganisms and fungi for fundamental research and biotechnology [42,43,44,45]. Aside from GeoCas9, a few examples include Cas9 from *Acidothermus cellulolyticus* (AceCas9), *Geobacillus thermodenitrificans T12* (ThermoCas9), *Ignavibacteriae phylum* (IgnaviCas9), and others. AceCas9 is used for gene editing in model thermophilic bacteria for biofuel processing. This nuclease is also found to be sensitive to the methylation state of the first but not the second cytosine base, making it a potential epigenomic detector [46,47,48]. ThermoCas9 has demonstrated its editing activity in model thermophilic organisms and human cells [45,49,50,51]. IgnaviCas9 retains its activity at temperatures up to 100 °C, which is proposed to be leveraged for the depletion of molecules derived from 16S rRNA in bacterial RNA-Seq libraries [52]. Further research and discoveries of novel thermophilic Cas9 orthologs will broaden the existing collection, thus expanding the application of the CRISPR-based tools (nickases, base editors, etc.) to multiple areas.

The results obtained demonstrate the reconstruction and in vitro characterization of yet another compact thermophilic Cas9 ortholog, AnoCas9. Although it shows high similarity to GeoCas9, there are also differences such as temperature range, which is slightly wider for GeoCas9, and the PAM sequence, which is slightly broader for AnoCas9. This distinguishes AnoCas9 as an individual nuclease, thereby adding to the repertoire of thermostable and thermophilic Cas effectors.

PAM recognition is a key stage for Cas9 activity, which determines targets available for CRISPR-mediated editing. Currently, studies on obtaining Cas9 variants with varying PAM preferences typically employ site-directed mutagenesis [53,54,55,56]. The characterization of abundant naturally occurring Cas9 nucleases, on the other hand, is beneficial not only for the expansion of the CRISPR toolkit but also for the structural studies on mechanisms of PAM recognition and cleavage initiation [57,58,59,60]. A few discovered thermophilic Cas9 proteins demonstrate varying stringency in PAM preferences (Table 1). AnoCas9 nuclease fits into this group as one of the smallest Cas9 effectors compared to SpyCas9 (1368 aa). It also demonstrates the highest similarity in PAM preference to ThermoCas9 and GeoCas9, which is consistent with the close relation of the *Anoxybacillus* genus to *Geobacillus*. Further investigations on thermophilic microorganisms in order to obtain novel thermostable Cas9 variants will be helpful for the development of a site-directed mutagenesis strategy to generate an effective thermostable Cas9 protein with relaxed PAM preferences or even PAMless editing ability.

While the usage of AnoCas9 for genome editing in human and mammalian cells, as well as thermophilic microorganisms, is clearly intriguing considering the similarity with GeoCas9, the experimental verification is yet to be performed, so it is more reasonable to focus on the possible applications in vitro. Given its working temperature range, AnoCas9, for example, could potentially be employed for allele-specific isothermal amplification as a helper nuclease cleaving the non-target allele template to prevent non-specific amplification [63,64,65]. Aside from the abovementioned diagnostic approaches involving thermophilic Cas9, the properties of such systems are also suggestive of their use for the DNA samples’ enrichment prior to the high-throughput target sequencing.

## 4. Materials and Methods

### 4.1. Materials

Gene amplification for the subsequent cloning was conducted using Q5 polymerase (NEB). Routine amplification and clone testing were performed using the BioMaster HS-Taq PCR-Color (2×) mix (Biolabmix Ltd., Novosibirsk, Russia). Sanger sequencing of the AnoCas9 ORF was carried out using the BigDye™ Terminator v3.1 Cycle Sequencing Kit (Thermo Fisher Scientific, Waltham, MA, USA), with products being further analyzed using the ABI 3130XL Genetic Analyser at the SB RAS Genomics Core Facility. Oligonucleotide primers were synthesized by Biosset Ltd. (Novosibirsk, Russia). Table 2 represents oligonucleotide sequences.

Cloning was performed using the NEBuilder HiFI DNA Assembly Master Mix kit (NEB). Plasmid restriction was performed using HindIII, XmaI, and KpnI endonucleases (SibEnzyme Ltd., Novosibirsk, Russia). Electrocompetent *E. coli* cells TOP10 or BL21 (DE3) were transformed using MicroPulser (Bio-Rad, Hercules, CA, USA). Plasmid DNA was isolated using the Plasmid DNA Isolation Maxi kit (Biolabmix Ltd., Russia). Bacterial strains and plasmids used in the study are presented in Table 3. Vector design, primer design, and sequencing analysis were conducted using the Unipro UGENE tool v.43 [66].

Chromatography was performed using the IMAC SepFast^TM^ and SP SepFastTM 6HF columns (Biotoolomics, Consett, UK). HMBP-Cas9 was cleaved using TEV-protease (Biolabmix Ltd., Russia). Proteins were analyzed using electrophoresis in the Mini-Protean Tetra System (Bio-Rad) and Precision Plus Protein Dual Color Standards (Bio-Rad) as markers.

The genome region encoding tracrRNA was amplified using the BioMaster HS-Taq PCR-Color (2×) mix (Biolabmix Ltd., Russia). Oligonucleotide primers were synthesized at the Laboratory of Synthetic Biology (ICBFM SB RAS, Novosibirsk, Russia). Table 4 represents oligonucleotide sequences. tracrRNA was transcribed in vitro using the high-yield T7 RNA synthesis kit (Biolabmix Ltd., Russia). DNA template and tracrRNA were isolated using the DR Reaction mixtures DNA isolation kit (Biolabmix Ltd., Russia).

The pCMV6 plasmid carrying the ANXA6 cDNA insert (pANXA6, OriGene #RC202086) was used in the Cas9 cleavage assays. The pMJ806 plasmid encoding *S. pyogenes* Cas9 (SpyCas9) carrying an N-terminal His6 tag, maltose-binding protein sequence, and TEV protease cleavage site was obtained from Addgene (#39312). SpyCas9 protein was overexpressed and purified according to a previously described protocol [67] for further usage as a reference Cas9 nuclease.

FAM-labeled double-stranded DNA (dsDNA) PAM templates were generated through routine amplification using the BioMaster HS-Taq PCR-Color (2×) mix (Biolabmix Ltd., Russia). Oligonucleotide primers were synthesized at the Laboratory of Synthetic Biology (ICBFM SB RAS). Table S1 represents oligonucleotide sequences. DNA templates were isolated using the DR Reaction mixtures DNA isolation kit (Biolabmix Ltd., Russia). Cas9 cleavage products were purified using the MagBlood DNA isolation kit (Biolabmix Ltd., Russia).

### 4.2. Whole Genome Sequencing and Data Analysis

A number of bacterial strains were selected from the Collection of Extremophilic Microorganisms and Type Cultures (Institute of Chemical Biology and Fundamental Medicine, Novosibirsk, Russia) and cultivated. Bacterial DNA was isolated and purified using DNA and RNA isolation and precipitation kit (Biolabmix, Novosibirsk, Russia) and used to construct libraries with TruSeq DNA PCR-Free kit (Illumina, San Diego, CA, USA) and TruSeq Illumina adaptors (Illumina). Synthesized libraries were sequenced on Illumina NovaSeq 6000 with NovaSeq 6000 S2 Reagent Kit v1.5 (2 × 150 cycles). Libraries were sequenced in biological duplicates, and raw data were demultiplexed with bcl2fastq v2.20.0.422 using default parameters. Four data archives were generated for each biological sample. Filtration by quality and adapter removal were performed with Trimmomatic v.0.39. Trimmed reads were then used to assemble genome contigs using SPADes v.3.15.4. All contigs longer than 500 bp were annotated for the presence of ORFs and CRISPR arrays with the RAST server.

### 4.3. Gene Sources and Cloning Strategy

*Anoxybacillus flavithermus* strain 4025 from the Collection of Extremophilic Microorganisms and Type Cultures (Institute of Chemical Biology and Fundamental Medicine, Novosibirsk) was selected as a model microorganism expressing thermophilic Cas9. Genomic DNA was used as a gene source.

The *anoCas9* gene was amplified using Cas9-4025_ext_F/R primers (Table 2). Oligos contained an overlap with a modified pET36b(+) vector [40]. *anoCas9* was cloned into the vector pre-digested with HindIII. Bacterial colonies containing pETm-Cas9 plasmids were selected using PCR with flanking primers 268F and 269R (Table 2), with the presence of elongated products (3566 bp) as the selection criterion.

Subsequently, the *anoCas9* gene was codon-optimized for the expression in *E. coli* using the http://atgme.org/ web tool (accessed on 12 September 2022) [68] and the *E. coli* strain W3110 [gbbct]: 4332 CDSs as a reference genome (1,372,057 codons). As a result, rare codons found in the first 210 bp from the 5′-end were replaced. The optimized DNA fragment was synthesized de novo in the Laboratory of Synthetic Biology (ICBFM SB RAS) and cloned into the pUC19 vector. The novel 5′-fragment of *anocas9* was used to assemble a partially optimized *anoCas9* gene and clone it into the expressing vector pD441-HMBP.

*anoCas9* was cloned into pD441-HMBP using KpnI and XmaI digestion sites. To amplify the optimized gene fragment (210 bp), 1F/2R primers were used (Table 2), containing an overlap with the pD441-HMBP vector and wild-type *anoCas9*. The remaining *anoCas9* fragment was amplified using 3F/4R primers (Table 2). The oligos were overlapping with the optimized 5′-fragment and pD441-HMBP. The NEBuilder kit was used to assemble three fragments—a hydrolyzed vector and two PCR products. Colonies were screened for the presence of the full-size product (3580 bp) using PCR with 5F/5R primers.

Finally, construct sequences were verified through the Sanger sequencing in the SB RAS Genomics Core Facility using Seq_Cas9(Anoav)_1-10 primers (Table 2).

### 4.4. Escherichia coli Cultivation Conditions

Genome engineering procedures involving *Escherichia coli* were conducted using the standard LB medium with an addition of kanamycin (30 μg/mL).

Biomass for protein isolation was cultivated using the corresponding vector and Rosetta 2 (DE3) as a production strain. Then, 100 mL of cell culture was incubated with kanamycin (30 μg/mL) and chloramphenicol (25 μg/mL) overnight at 37 °C, 200 rpm. The next day, 7.5 mL of inoculate was transferred into 2.5 L Ultra Yield Thomson flasks (Thomson Ltd., Leeds, UK) containing 750 mL of fresh ZYM-505 media (no microelements or lactose addition) [69], with the abovementioned antibiotics added. Cells were cultivated at 37 °C, 200 rpm in the Excella E25 incubator shaker (Eppendorf, Hamburg, Germany) to reach the OD600 of 0.6–0.8 o.u./mL (total media volume equaled 6 L, divided into 8 flasks). Next, 0.4 mL of 1 M isopropyl β-D-1-thiogalactopyranoside (IPTG) was added into flasks to reach the final concentration of ~0.5 mM. Afterward, cells were cultivated for 18 h at 20 °C, 200 rpm. Cells were harvested via centrifugation for 20 min at ~10,000× *g*, 4 °C (Hitachi CR22GIII, R9A rotor). The biomass was stored at −80 °C until protein isolation.

### 4.5. AnoCas9 Purification

#### 4.5.1. Collection of the Cell Extract

Biomass (25 ± 5 g) was resuspended in 250 mL of Buffer A (50 mM Tris-HCl (pH 8.0 at 25 °C); 300 mM NaCl), adding PMSF to the final concentration of 1 mM. Cell suspension was placed into the ice bath and sonicated for 1 min three times (with pauses for 2 min). Cell debris was pelleted for 30 min at ~32,000× *g*, 4 °C (Hitachi CR22GIII, R19A rotor), and the supernatant was used for chromatography.

#### 4.5.2. IMAC-Sepharose Chromatography

Immobilized metal affinity chromatography (IMAC) was used as the first step of protein purification. The supernatant was applied to the column with a resin that had been equilibrated in advance with Buffer A, flowing at a speed of 1–2 mL/min. The column was then washed with 100 mL of Buffer A. The target precursor protein HMBP-AnoCas9 was eluted with a linear imidazole gradient (0–300 mM). The gradient was prepared using Buffers A and B (50 mM Tris-HCl (pH 8.0 at 25 °C); 300 mM NaCl, 300 mM imidazole), so that the volume ratio of Buffer B increased from 0% to 100% within three volumes of resin. Collected fractions were analyzed for the presence of the HMBP-Cas9 precursor protein. Next, the purest positive fractions were combined, and the A280 adsorption was measured to assess protein concentration. The HMBP-AnoCas9 protein was treated with TEV-protease at ~1000 u per 50 mg of protein ratio. The reaction mixture was transferred into a dialysis bag to perform dialysis at 4 °C overnight (buffer contained 20 mM Tris-HCl (pH 8.0 at 25 °C), 100 mM NaCl, 1 mM EDTA, 1 mM dithiothreitol, 10% glycerol). The next day, the reaction mixture was centrifuged for 30 min at ~32,000× *g*, 4 °C, and the supernatant was used for SP-Sepharose purification.

#### 4.5.3. SP-Sepharose Chromatography

The supernatant was applied to the resin column (volume 15 mL) pre-equilibrated with Buffer C (20 mM Tris-HCl (pH 8.0 at 25 °C), 100 mM NaCl) at a 1–2 mL/min speed. The column was subsequently washed with 80 mL of Buffer A. The target AnoCas9 was eluted with a sodium chloride linear gradient (100–1000 mM). The gradient was prepared using Buffers C and D (20 mM Tris-HCl (pH 8.0 at 25 °C); 1 M NaCl) so that the volume ratio of Buffer D increased from 0% to 100% within three volumes of the resin. Collected fractions were analyzed for the presence of the AnoCas9 protein. The purest positive fractions were combined and concentrated at 4000 rpm, 4 °C, to the final volume of 0.5–1.0 mL using the Eppendorf Centrifuge 5810R. Then, 20 mL of the conservation buffer (20 mM Tris-HCl (pH 7.4 at 25 °C), 600 mM NaCl, 0.2 mM EDTA, 2 mM dithiothreitol) was added, and the concentration procedure was repeated. Finally, the concentrate was transferred into a clean Eppendorf tube, and an equal volume of glycerol was added dropwise. For the final solution, electrophoretic purity was determined, OD280 measured, and AnoCas9 concentration assessed (1 o.u. was considered equal to 1.17 mg/mL or 9.2 pmol/µL). The protein was stored at −20 °C in the final solution containing 10 mM Tris-HCl (pH 7.4 at 25 °C), 300 mM NaCl, 0.1 mM EDTA, 1 mM dithiothreitol, and 50% glycerol. Proteins and cell lysates were visualized via 10–12% denaturing SDS-PAGE with Coomassie Brilliant Blue G-250 staining.

### 4.6. CRISPR Array Analysis

AnoCas9 nucleotide and amino acid sequences were analyzed using BLAST alignment tools. Whole-genome sequencing data were analyzed using the RAST server to determine the corresponding CRISPR array. Spacer sequences were annotated and further used to predict the PAM sequence using the CRISPRTarget web tool [70]. Spacer sequences were aligned with the data from Genbank-Phage, RefSeq-plasmid, and IMGVR databases. The highest-scoring alignments were further analyzed to determine a set of putative PAM motifs. Data were combined using WebLogo v. 3.7.11 [71].

### 4.7. CRISPR RNA Synthesis

Genomic DNA was used as a template to amplify the tracrRNA-encoding region. A short dsDNA template was isolated and used subsequently to generate tracrRNA through in vitro transcription. CRISPR RNAs (crRNAs) with varying spacer sequences were synthesized de novo in the Laboratory of Synthetic Biology (ICBFM SB RAS).

### 4.8. In Vitro DNA Plasmid Cleavage with Cas9

In vitro cleavage assays were performed as described [41]. Prior to the experiment, crRNA was hybridized with tracrRNA (2 μM each) in deionized water at 95 °C for 30 s followed by 3 min on ice. SpyCas9 or AnoCas9 ribonucleoprotein (RNP) complex was assembled (final concentrations: 0.4 μM Cas9 (Sp or Ano), 2 μM tracrRNA, and 2 μM crRNA) in a reaction buffer (20 mM HEPES pH 7.5, 100 mM KCl, 5% glycerol, 1 mM DTT, 0.5 mM EDTA, 2 mM MgCl2) at 37 °C for 20 min. The target pANXA6 plasmid was cleaved in the presence of a 50-fold excess of Cas9/crRNA:tracrRNA and a 1:5 molar ratio of Cas9 to the crRNA:tracrRNA complex. Samples were incubated at the indicated temperatures (37–75 °C) for 1 h and then quenched by adding 10 µL of proteinase K (60 mM EDTA, 4 M urea, 0.4 mg/mL proteinase K) and incubating at 37 °C for 15 min. Products were resolved through the electrophoresis in 1% agarose gel, stained with 0.5 µg/mL ethidium bromide, and visualized using a UV imager.

The images of gel electrophoresis were quantified using Quantity One 4.6.8 (Bio-Rad Laboratories, Hercules, CA, USA). The percentages of single- and double-strand breaks of plasmid (SSB and DSB, respectively) were calculated as:

$$efficiency,\%=\frac{V_{l}}{V_{r}+V_{l}+V_{sc}\cdot k}\cdot 100\%$$

where V is the intensity of bands corresponding to the relaxed (V_r_), linear (V_l_), and supercoiled (V_sc_) plasmids; and k = 1.14 is the ethidium bromide intercalation coefficient in the supercoiled plasmid [41].

### 4.9. In Vitro PAM Library Digestion with Cas9

Rapid identification of functional PAM motifs for AnoCas9 was performed through the cleavage of a fluorescently labeled dsDNA fragment library. The method adapts the standard approach using radiolabeled dsDNA substrates [72]. A set of oligonucleotides, containing sequences complementary to the spacer and variable nucleotides at different positions in the PAM motif, was synthesized in the Laboratory of Synthetic Biology ICBFM SB RAS (referred to as Spacer-PAM-RN, Table S1). The single-stranded template was turned into dsDNA using the second universal oligonucleotide carrying the spacer sequence at its 3′-end and thus complementary to the Spacer-PAM-RN 3′-end (Uni-Spacer-F, Table S1). Partial oligonucleotide duplexes were elongated through routine three-step amplification. PCR products were then utilized as templates to generate elongated FAM-labeled dsDNA substrates using a Uni-Spacer-F oligo and a universal 5′-FAM-labeled oligonucleotide extending the generated dsDNA template downstream of PAM motif (FAM-Uni-R, Table S1). Three-step amplification allowed us to produce the final library of dsDNA substrates containing the upstream extension, spacer sequence, PAM motif of choice, downstream extension, and FAM label. DNA templates were isolated, OD260 was measured, and dsDNA concentration was assessed.

In vitro cleavage assays were performed as described in Section 4.8. Briefly, the cleavage of the target dsDNA template was carried out in the presence of a 25-fold excess of Cas9/crRNA:tracrRNA and a 1:5 molar ratio of Cas9 to crRNA:tracrRNA complex. The samples were incubated at 50 °C for 1 h and then quenched by adding proteinase K. The fragments were purified and further resolved using the Nanophore 05 gene analyzer (Syntol, Moscow, Russia). The ratio of cleaved DNA to the total dsDNA amount was assessed and used to quantify editing efficiency.

## 5. Conclusions

The current study presents the procedure for the reconstruction and expression of a thermophilic Cas9 ortholog from *Anoxybacillus flavithermus* in *E. coli* cells. The AnoCas9 protein exhibited nuclease activity in vitro at temperatures up to 60 °C and PAM preference of 5′-NNNNCDAA-3′, fitting into the family of thermophilic *Geobacillus* Cas9 variants. Previously varying diagnostic approaches requiring the presence of thermophilic Cas9 had been described; hence, we strongly believe that the characterized PAM preference for AnoCas9 will soon allow for developing the analogous tool.

## Figures and Tables

**Table 1:** Most prominent thermophilic Cas9 nucleases, their sources, and PAM preferences.

Name	Source	Size, aa	PAM Sequence (5′-3′)	References
ThermoCas9	*Geobacillus thermodenitrificans T12*	1082	NNNNCVAA NNNNCCCA	[49]
GeoCas9	*Geobacillus stearothermophilus*	1087	NNNNCRAA	[35]
CaldoCas9	*Geobacillus* sp. *LC300*	1087	NNNNGNMA	[61]
AceCas9	*Acidothermus cellulolyticus*	1138	NNNCC	[46,47]
IgnaviCas9	*Ignavibacteriae phylum*	1241	NRRNAT	[52]
AtCas9	*Alicyclobacillus tengchongensis*	1147	NNNNCNNN NNNNRNNA	[62]
AnoCas9	*Anoxybacillus flavithermus*	1087	NNNNCDAA	

**Table 2:** Amplification and sequencing primers used for cloning.

Name	Structure (5′–3′)
Cas9-4025_ext_F	cgcagcggtcacccgaagcttatgagatataaaataggattagacatag
Cas9-4025_ext_R	tggtggtgctcgagcagaagcttatgactaattgattgtaacgaatg
1F	cgaaaacctgtattttcagggcggtaccatgcgttataaaattggtttagacatc
2R	cgttaaaacaccttcacggactaaaagacggcgaatacgttctaaacgatgtttg
3F	cgtcttttagtccgtgaaggtg
4R	tcagtcgaaagactgggcctttcgcccgggctaatttcaatgactaattgattgtaacgaatg
268F	atgcgtccggcgtaga
269R	gctagttattgctcagcggtg
5F	agactgtcgatgaagccctg
5R	tgccgaactcagaagtgaaac
Seq_Cas9(Anoav)_1	actccatcgcagaagctgc
Seq_Cas9(Anoav)_2	gcaaatgtcttgaacaagtttatgg
Seq_Cas9(Anoav)_3	cgtcttttagtccgtgaaggtg
Seq_Cas9(Anoav)_4	ccagctgagtatttaggattcg
Seq_Cas9(Anoav)_5	ggtccaatcattcgtactgtg
Seq_Cas9(Anoav)_6	cagtaccgttacaccataattcac
Seq_Cas9(Anoav)_7	acgcatcaacacgcaactgc
Seq_Cas9(Anoav)_8	ctctttgaattcttgttcttctgc
Seq_Cas9(Anoav)_9	caagttctttttccatcagcacg
Seq_Cas9(Anoav)_10	tcaaaaacatcggtgcgaacg

**Table 3:** Strains and plasmids used to generate the construct expressing AnoCas9.

Strains	Relevant Genotype	Source
*Anoxybacillus flavithermus*	Wild type	CEMTC (ICBFM SB RAS)
*E. coli* TOP10	F– mcrA Δ(mrr-hsdRMS-mcrBC) φ80lacZΔM15 ΔlacX74 recA1 araD139 Δ(ara-leu)7697 galU galK rpsL endA1 nupG	Thermo Scientific™ (Waltham, MA, USA)
*E. coli* BL21 (DE3)	F– ompT hsdSB (rB–, mB–) gal dcm (DE3)	Thermo Scientific™
*E. coli* Rosetta 2 (DE3)	F– ompT hsdSB (rB–, mB–) gal dcm (DE3) pRARE2 (Cam^R^)	Novagen (Sigma-Aldrich, St. Louis, MO, USA)
**Plasmids**	**Description**	**Source**
pET36b(+)	PT7lac, Kan^R^	Novagen
pETm-Cas9	PT7lac, Kan^R^	This study
pUC19-210Cas9	pBR233ori-F, Amp^R^	ICBFM SB RAS
pD441-HMBP	PT5lac, Kan^R^	DNA TwoPointO Inc. (Menlo Park, CA, USA) («ATUM»)
pD441-HMBP-Cas9	PT5lac, Kan^R^	This study

**Table 4:** Amplification primers used to generate the dsDNA template and crRNA sequences.

Name	Structure (5′-3′)
Ano_tracrRNA_T7	atgcagctaatacgactcactataggtcagggttactatgataagg
Ano_tracrRNA_R	atggggagtgcccttaaagg
Ano_ANXA_cr	cagggaugcauuuguggccauguaauaguuccccuga

## Data Availability

All data generated or analyzed during this study are included in this article.

## Supplementary Materials

The following supporting information can be downloaded at: https://www.mdpi.com/article/10.3390/ijms242317121/s1.
