# Supplementary material for: Expression and Functional Analysis of the Compact Thermophilic Anoxybacillus flavithermus Cas9 Nuclease
Source: Int J Mol Sci. 2023 Dec 4;24(23):17121. doi: 10.3390/ijms242317121 (PMC10707453; doi:10.3390/ijms242317121)
Supplement: Supplementary file 1 [file ijms-24-17121-s001.zip › Supplementary/Supplementary Figures and Tables.pdf]

Supplementary Materials:

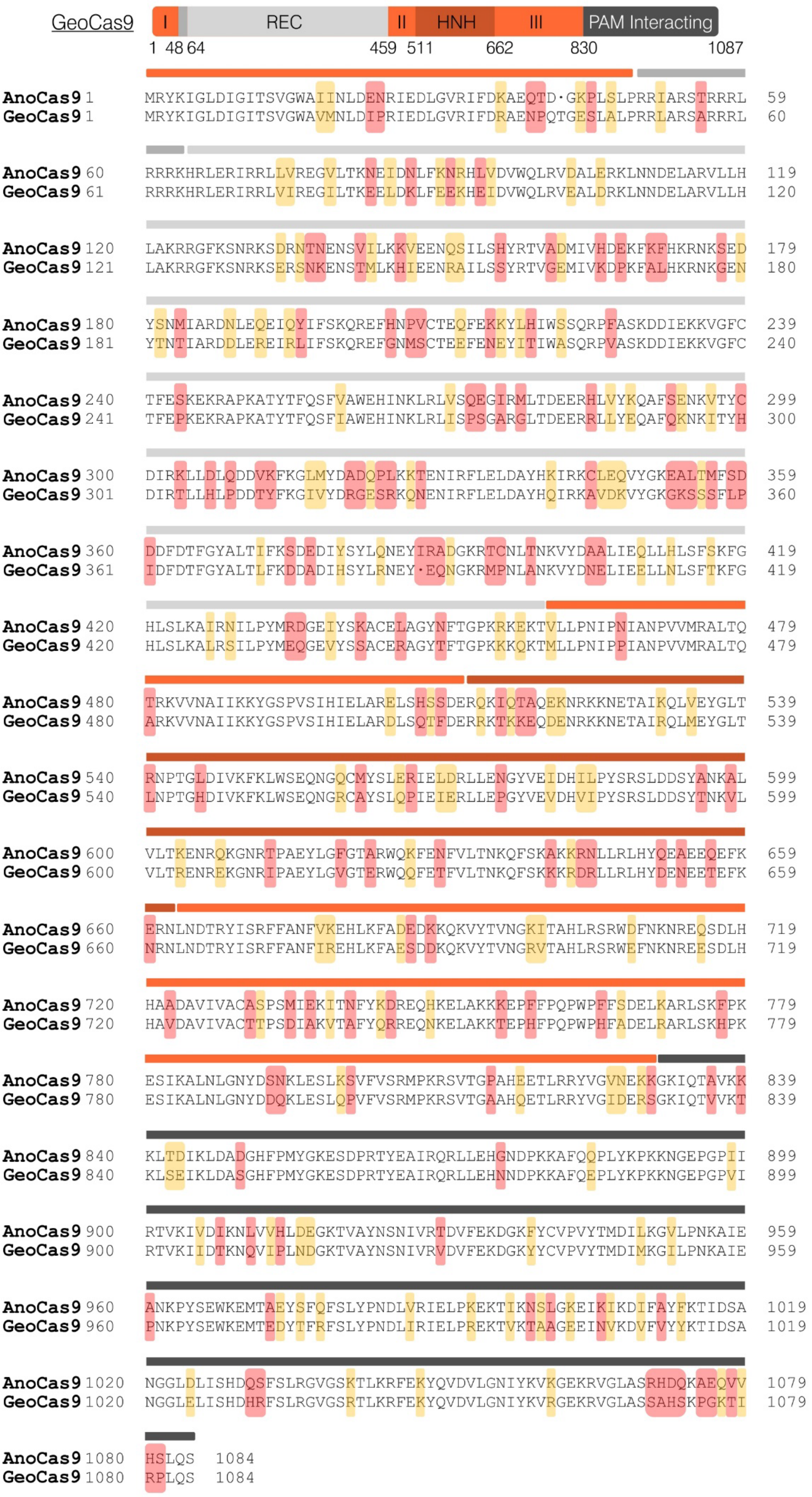

Figure S1. Alignment of AnoCas9 and GeoCas9 protein sequences. Mismatches are highlighted in red and conservative substitutions are highlighted in yellow. GeoCas9 domain structure is presented with domains marked with varying colors.

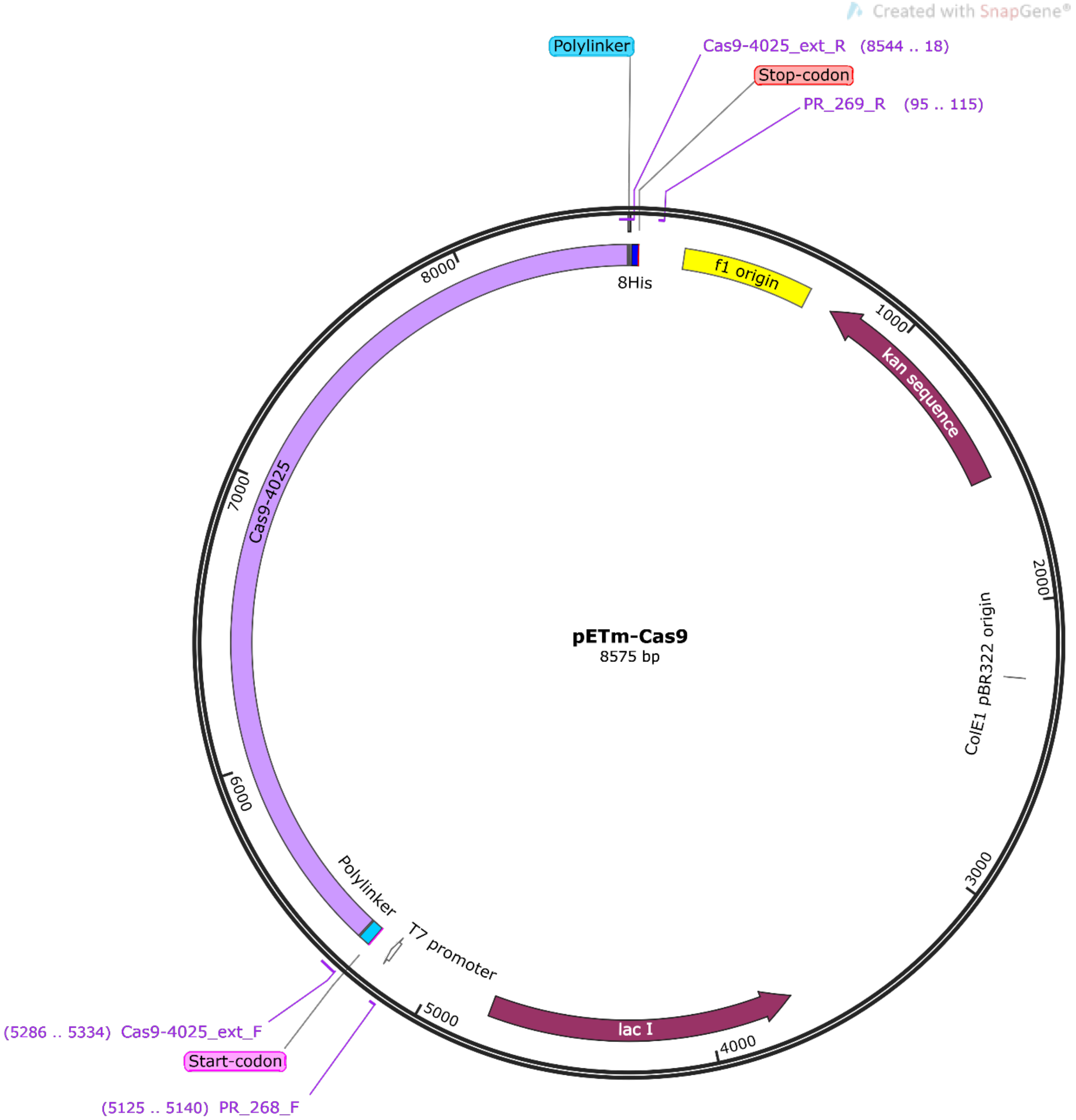

Figure S2. pETm-AnoCas9 map.

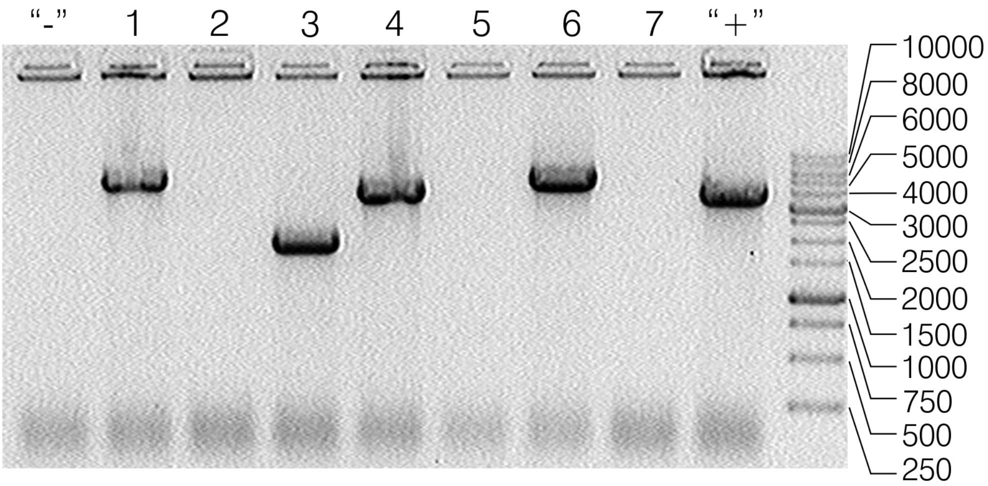

Figure S3. Analysis of PCR products after electrophoresis in 1% agarose gel. The expected product length was 3,580 bp in the case of the *anoCas9* insertion. Lane "-" – negative control, lane "+" – positive control (ligation mixture was used as a template). Lanes 1-7 refer to the colonies 1-7, respectively.

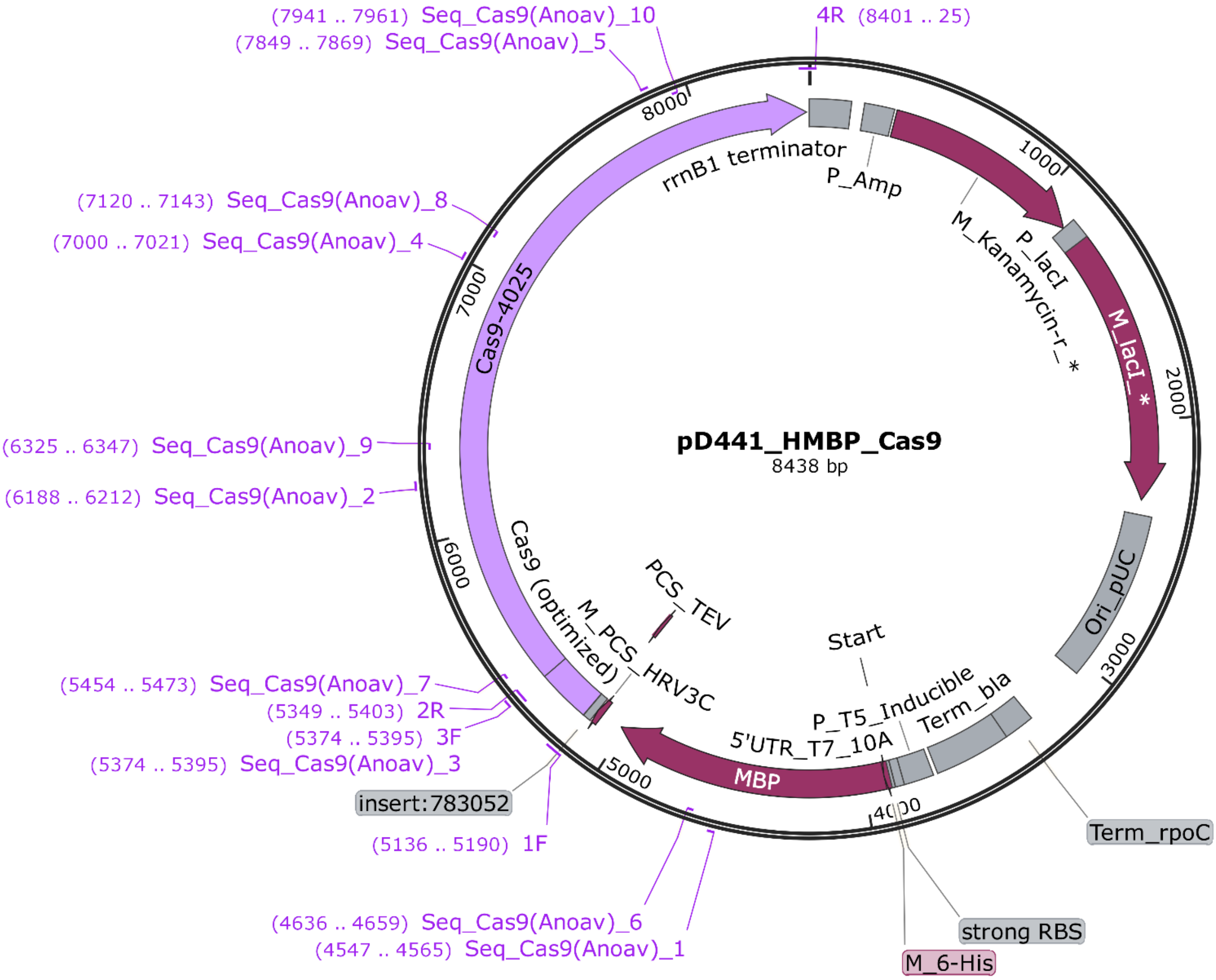

Figure S4. pD441-HMBP-AnoCas9 map.

```
1. Match to: (IMGVR_UViG_2619618815_000002|2619618815|2619619304|78227-107433 position: 11709-11738,
with: spacer13 CRISPR No.13 Spacer No.1 position: 1-30, Strand: -, Direct Repeat: , Type[cctyper]:
5' --------CAGAAAGGGUUGGAAGGUGAUUCGAUGAAG-------- 3'    <- CRISPR spacer RNA
3' TCAGCATAGTCTTTCCCTACCTTCCACTAAGCTACTTCTTCAGTTG 5'    <- Protospacer Sequence
5' AGTCGTATCAGAAAGGGATGGAAGGTGATTCGATGAAGAAGTCAAC 3'    <- [JGI Link (UViG=IMGVR_UViG_2619618815_000002)]
   Score: 28
```

```
2. Match to: (IMGVR_UViG_2642422507_000003|2642422507|2642424256|76224-104615) position: 11325-11354,
with: spacer13 CRISPR No.13 Spacer No.1 position: 1-30, Strand: -, Direct Repeat: , Type[cctyper]:
5' --------CAGAAAGGGUUGGAAGGUGAUUCGAUGAAG-------- 3'    <- CRISPR spacer RNA
3' TCGGCATAGTCTTTCCCTACCTTCCACTAAGCTACTTCTTTGGCTT 5'    <- Protospacer Sequence
5' AGCCGTATCAGAAAGGGATGGAAGGTGATTCGATGAAGAAACCGAA 3'    <- [JGI Link (UViG=IMGVR_UViG_2642422507_000003)]
   Score: 28
```

```
3. Match to: (IMGVR_UViG_2808606689_000002|2808606689|2808643806|2434631-2475229) position: 25371-25342,
with: spacer13 CRISPR No.13 Spacer No.1 position: 1-30, Strand: +, Direct Repeat: , Type[cctyper]:
5' --------CAGAAAGGGUUGGAAGGUGAUUCGAUGAAG-------- 3'    <- CRISPR spacer RNA
3' TCGGCATAGTCTTTCCCGACCTTCCACTAAGCTACTTCTTCAGCTG 5'    <- Protospacer Sequence
5' AGCCGTATCAGAAAGGGCTGGAAGGTGATTCGATGAAGAAGTCGAC 3'    <- [JGI Link (UViG=IMGVR_UViG_2808606689_000002)]
   Score: 28
```

```
4. Match to: (IMGVR_UViG_2784746787_000001|2784746787|2784764528|7903-43494) position: 35213-35184,
with: spacer3 CRISPR No.3 Spacer No.1 position: 1-30, Strand: +, Direct Repeat: , Type[cctyper]:
5' --------AAAAAGGAGAUCAUGAAAAAAAGCAGUAGCU-------- 3'    <- CRISPR spacer RNA
3' GTTACTTGTTTTTCTCTAGTACTTTTTTCGTCAACGAAATCGCTT 5'    <- Protospacer Sequence
5' CAATGAACAAAAAGAGATCATGAAAAAAGCAGTTGCTTTAGCGAA 3'    <- [JGI Link (UViG=IMGVR_UViG_2784746787_000001)]
   Score: 26
```

```
5. Match to: (IMGVR_UViG_2861196614_000001|2861196614|2861196616|138535-188168) position: 21916-21944,
with: spacer3 CRISPR No.3 Spacer No.1 position: 1-30, Strand: -, Direct Repeat: , Type[cctyper]:
5' --------AAAAAGGAGAUCAUGAAAAAAAGCAGUAGCU-------- 3'    <- CRISPR spacer RNA
3' TTTACTTGTTTTTCTCTAGTACTTTTTTCGTCAACGTAATCGTTT 5'    <- Protospacer Sequence
5' AAATGAACAAAAAGAGATCATGAAAAAAGCAGTTGCATTAGCAAA 3'    <- [JGI Link (UViG=IMGVR_UViG_2861196614_000001)]
   Score: 24
```

```
6. Match to: (IMGVR_UViG_2861217274_000001|2861217274|2861217277|76193-111870) position: 21354-21327,
with: spacer13 CRISPR No.13 Spacer No.1 position: 1-30, Strand: +, Direct Repeat: , Type[cctyper]:
5' --------CAGAAAGGGUUGGAAGGUGAUUCGAUGAAG-------- 3'    <- CRISPR spacer RNA
3' TCGGCTTAAGCTTTTCCAACCTTCCACTAAGCTACTTCTTCGGCTC 5'    <- Protospacer Sequence
5' AGCCGAATTCGAAAAGGTTGGAAGGTGATTCGATGAAGAAGCCGAG 3'    <- [JGI Link (UViG=IMGVR_UViG_2861217274_000001)]
   Score: 24
```

```
7. Match to: (IMGVR_UViG_2576861798_000001|2576861798|2576924771) position: 19643-19616,
with: spacer13 CRISPR No.13 Spacer No.1 position: 1-30, Strand: +, Direct Repeat: , Type[cctyper]:
5' --------CAGAAAGGGUUGGAAGGUGAUUCGAUGAAG-------- 3'    <- CRISPR spacer RNA
3' AATTCATAGTCTTTCCCGACCTACCACTAAGCTACTACTCCTTTGT 5'    <- Protospacer Sequence
5' TTAAGTATCAGAAAGGGCTGGATGGTGATTCGATGATGAGGAAACA 3'    <- [JGI Link (UViG=IMGVR_UViG_2576861798_000001)]
   Score: 24
```

```
8. Match to: (IMGVR_UViG_2861220268_000001|2861220268|2861220269|23777-59174) position: 23109-23082,
with: spacer13 CRISPR No.13 Spacer No.1 position: 1-30, Strand: +, Direct Repeat: , Type[cctyper]:
5' --------CAGAAAGGGUUGGAAGGUGAUUCGAUGAAG-------- 3'    <- CRISPR spacer RNA
3' TCGGCTTAAGCTTTTCCAACCTTCCACTAAGCTACTTCTTCGGCTC 5'    <- Protospacer Sequence
5' AGCCGAATTCGAAAAGGTTGGAAGGTGATTCGATGAAGAAGCCGAG 3'    <- [JGI Link (UViG=IMGVR_UViG_2861220268_000001)]
   Score: 24
```

Figure S5. CRISPRTarget spacer analysis. First 8 targets are presented that were used to predict and create the PAM sequence logo.

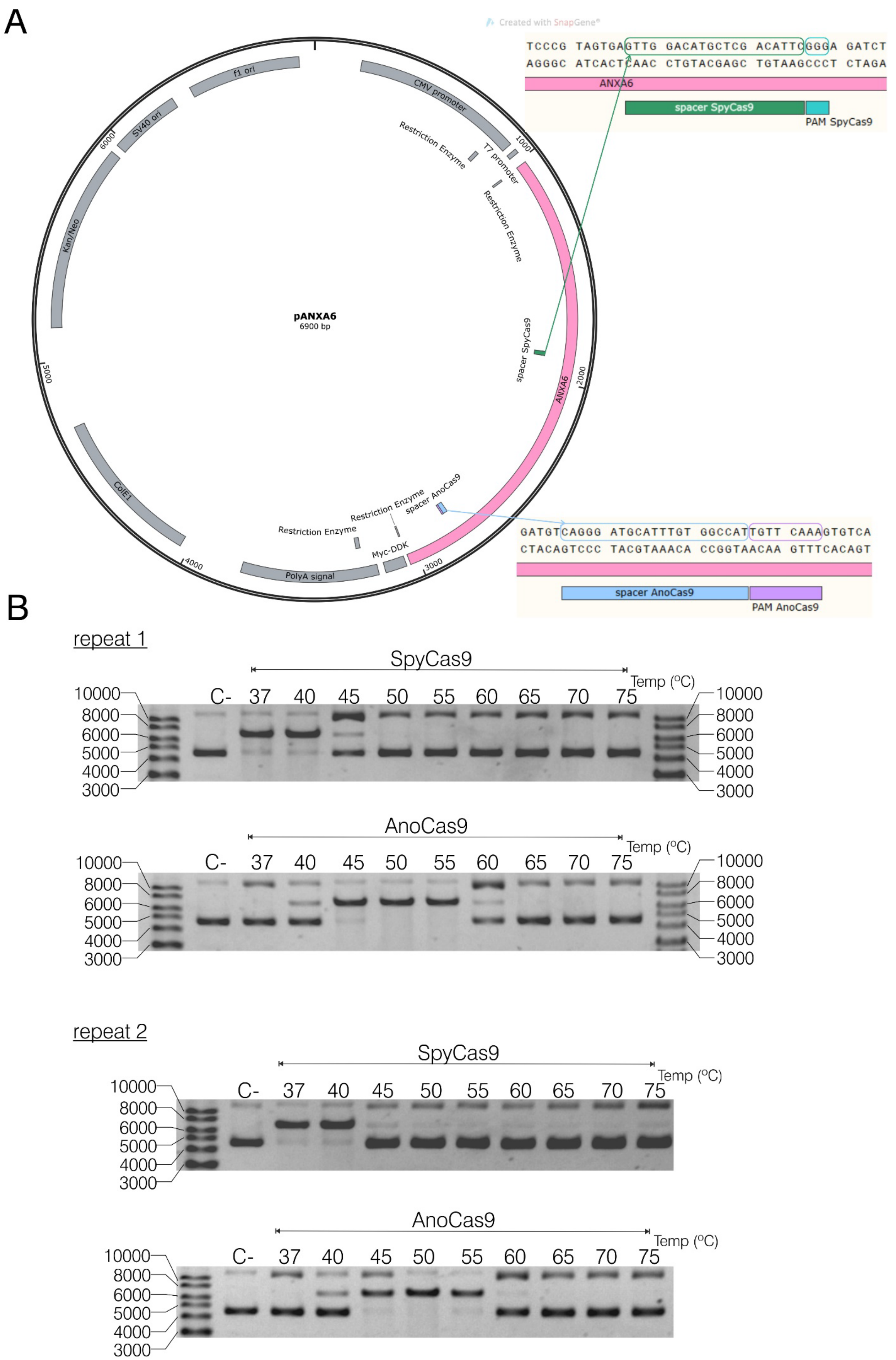

Figure S6. Experimental design for the Cas9 cleavage assay. The map of pANXA6 is presented with the spacer and PAM sequences for SpyCas9 and AnoCas9 highlighted (A). Technical replicates of the temperature gradient cleavage assay are presented (B). Reaction mixtures were resolved in the 1% agarose gel after incubation at the indicated temperature and quenching. Plasmid DNA without Cas9 treatment (C-) was used as a negative control.

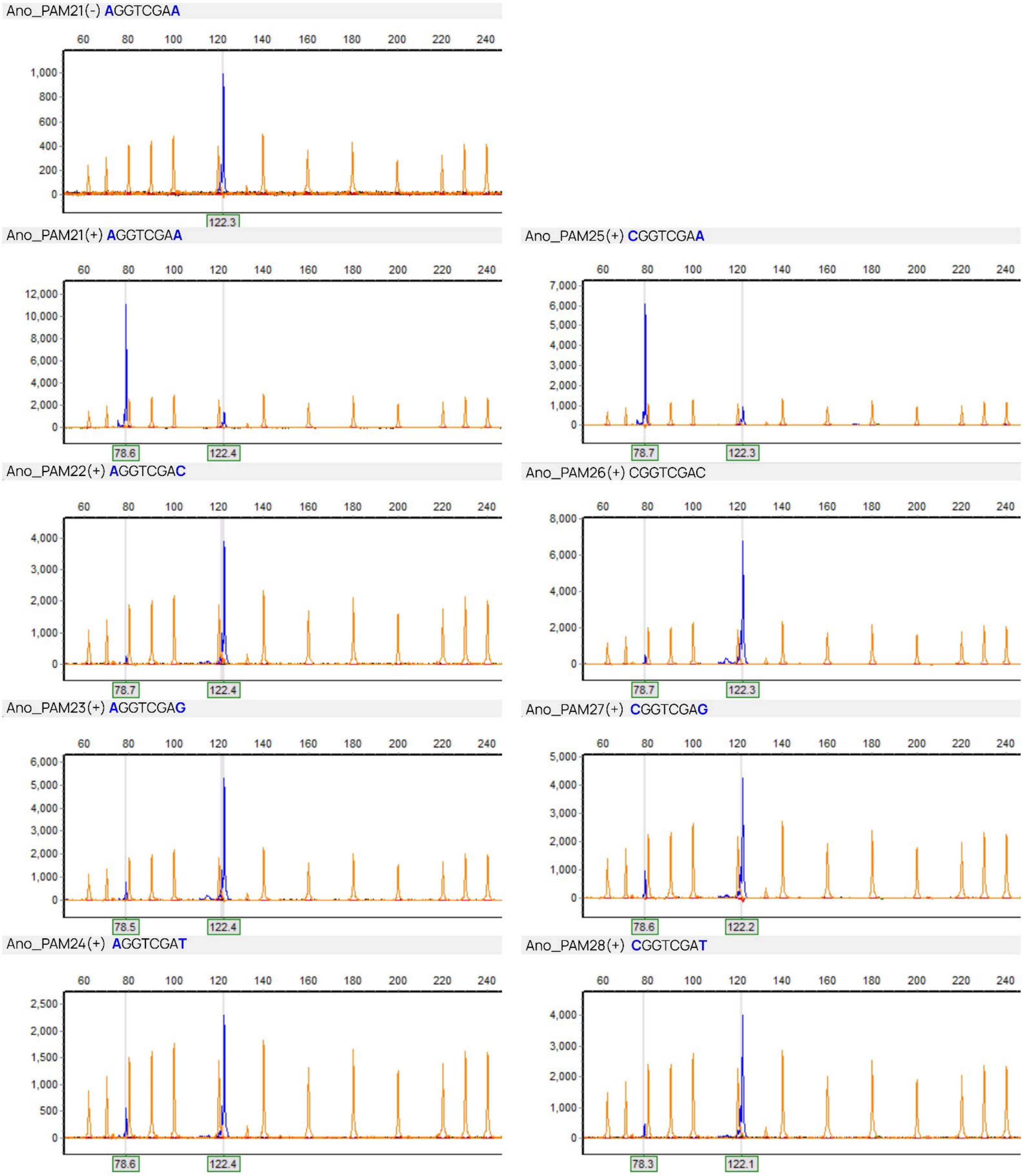

Figure S7. A few examples of electropherograms of the AnoCas9 digestion for the FAM-labeled PAM-variable DNA substrates. PAM (-) control refers to the sample with no Cas9 nuclease added. Variable PAM nucleotide positions for the displayed subset of substrates are marked in blue. The observed difference in the lengths of uncut and cut fragments compared to the expected ones from the experiment design is related to the overall short length of the substrates tested and seemingly lowered resolution of the gel analyzer in the selected area.

Table S1. Amplification primers used for the generation of FAM-labeled dsDNA PAM-templates.

Name	Structure (5′-3′)
Uni-Spacer-F	caagaaggagatgtctggggatgtcagggatgcatttgtggccat
FAM-Uni-R	ttctcatctgtgccagcacccttcatggatttgtaaagtttgtcggcaaagaagaga
Spacer-PAM-R_1	ttgtcggcaaagaagagaggcttgttcttgacactttgaacaatggccacaaatgcatc
Spacer-PAM-R_2	ttgtcggcaaagaagagaggcttgttcttgacacttggaacaatggccacaaatgcatc
Spacer-PAM-R_3	ttgtcggcaaagaagagaggcttgttcttgacacttcgaacaatggccacaaatgcatc
Spacer-PAM-R_4	ttgtcggcaaagaagagaggcttgttcttgacacttagaacaatggccacaaatgcatc
Spacer-PAM-R_5	ttgtcggcaaagaagagaggcttgttcttgacactttgatcaatggccacaaatgcatc
Spacer-PAM-R_6	ttgtcggcaaagaagagaggcttgttcttgacacttggatcaatggccacaaatgcatc
Spacer-PAM-R_7	ttgtcggcaaagaagagaggcttgttcttgacacttcgatcaatggccacaaatgcatc
Spacer-PAM-R_8	ttgtcggcaaagaagagaggcttgttcttgacacttagatcaatggccacaaatgcatc
Spacer-PAM-R_9	ttgtcggcaaagaagagaggcttgttcttgacactttgagcaatggccacaaatgcatc
Spacer-PAM-R_10	ttgtcggcaaagaagagaggcttgttcttgacacttggagcaatggccacaaatgcatc
Spacer-PAM-R_11	ttgtcggcaaagaagagaggcttgttcttgacacttcgagcaatggccacaaatgcatc
Spacer-PAM-R_12	ttgtcggcaaagaagagaggcttgttcttgacacttagagcaatggccacaaatgcatc
Spacer-PAM-R_13	ttgtcggcaaagaagagaggcttgttcttgacactttgaccaatggccacaaatgcatc
Spacer-PAM-R_14	ttgtcggcaaagaagagaggcttgttcttgacacttggaccaatggccacaaatgcatc
Spacer-PAM-R_15	ttgtcggcaaagaagagaggcttgttcttgacacttcgaccaatggccacaaatgcatc
Spacer-PAM-R_16	ttgtcggcaaagaagagaggcttgttcttgacacttagaccaatggccacaaatgcatc
Spacer-PAM-R_18	ttgtcggcaaagaagagaggcttgttcttgacacgtcgaccaatggccacaaatgcatc
Spacer-PAM-R_19	ttgtcggcaaagaagagaggcttgttcttgacacctcgaccaatggccacaaatgcatc
Spacer-PAM-R_20	ttgtcggcaaagaagagaggcttgttcttgacacatcgaccaatggccacaaatgcatc
Spacer-PAM-R_21	ttgtcggcaaagaagagaggcttgttcttgacacttcgacctatggccacaaatgcatc
Spacer-PAM-R_22	ttgtcggcaaagaagagaggcttgttcttgacacgtcgacctatggccacaaatgcatc
Spacer-PAM-R_23	ttgtcggcaaagaagagaggcttgttcttgacacctcgacctatggccacaaatgcatc
Spacer-PAM-R_24	ttgtcggcaaagaagagaggcttgttcttgacacatcgacctatggccacaaatgcatc
Spacer-PAM-R_25	ttgtcggcaaagaagagaggcttgttcttgacacttcgaccgatggccacaaatgcatc
Spacer-PAM-R_26	ttgtcggcaaagaagagaggcttgttcttgacacgtcgaccgatggccacaaatgcatc
Spacer-PAM-R_27	ttgtcggcaaagaagagaggcttgttcttgacacctcgaccgatggccacaaatgcatc
Spacer-PAM-R_28	ttgtcggcaaagaagagaggcttgttcttgacacatcgaccgatggccacaaatgcatc
Spacer-PAM-R_29	ttgtcggcaaagaagagaggcttgttcttgacacttcgacccatggccacaaatgcatc
Spacer-PAM-R_30	ttgtcggcaaagaagagaggcttgttcttgacacgtcgacccatggccacaaatgcatc
Spacer-PAM-R_31	ttgtcggcaaagaagagaggcttgttcttgacacctcgacccatggccacaaatgcatc
Spacer-PAM-R_32	ttgtcggcaaagaagagaggcttgttcttgacacatcgacccatggccacaaatgcatc
Spacer-PAM-R_33	ttgtcggcaaagaagagaggcttgttcttgacacttcgtcttatggccacaaatgcatc
Spacer-PAM-R_34	ttgtcggcaaagaagagaggcttgttcttgacacttcgtcgtatggccacaaatgcatc
Spacer-PAM-R_35	ttgtcggcaaagaagagaggcttgttcttgacacttcgtcctatggccacaaatgcatc
Spacer-PAM-R_36	ttgtcggcaaagaagagaggcttgttcttgacacttcgtcatatggccacaaatgcatc
Spacer-PAM-R_37	ttgtcggcaaagaagagaggcttgttcttgacacttcggcttatggccacaaatgcatc
Spacer-PAM-R_38	ttgtcggcaaagaagagaggcttgttcttgacacttcggcgtatggccacaaatgcatc
Spacer-PAM-R_39	ttgtcggcaaagaagagaggcttgttcttgacacttcggcctatggccacaaatgcatc
Spacer-PAM-R_40	ttgtcggcaaagaagagaggcttgttcttgacacttcggcatatggccacaaatgcatc
Spacer-PAM-R_41	ttgtcggcaaagaagagaggcttgttcttgacacttcgccttatggccacaaatgcatc
Spacer-PAM-R_42	ttgtcggcaaagaagagaggcttgttcttgacacttcgccgtatggccacaaatgcatc
Spacer-PAM-R_43	ttgtcggcaaagaagagaggcttgttcttgacacttcgccctatggccacaaatgcatc
Spacer-PAM-R_44	ttgtcggcaaagaagagaggcttgttcttgacacttcgccatatggccacaaatgcatc
Spacer-PAM-R_45	ttgtcggcaaagaagagaggcttgttcttgacacttcgacttatggccacaaatgcatc
Spacer-PAM-R_46	ttgtcggcaaagaagagaggcttgttcttgacacttcgacgtatggccacaaatgcatc
Spacer-PAM-R_48	ttgtcggcaaagaagagaggcttgttcttgacacttcgacatatggccacaaatgcatc

Table S2. Sequences of CRISPR array elements derived from the *Anoxybacillus flavithermus* genome.

Name	Structure (5′-3′)
CRISPR repeat	gtaatagttcccctgaggttattgctgtgttatgat
CRISPR spacer 1	aaaatgacacacttatttgataaaaagaaa
CRISPR spacer 2	ccgaaagttttagactcactataaatcctc
CRISPR spacer 3	aaaaaggagatcatgaaaaaagcagtagct
CRISPR spacer 4	cgtgggtcaattcggtaaatggaagttttt
CRISPR spacer 5	tgctacacttttgcaccgaaacggagcgga
CRISPR spacer 6	agaccagtccaactagaaattaattttaga
CRISPR spacer 7	tgaagaaaaagaaaactgtagagaaacaac
CRISPR spacer 8	tcatcagcaaaaatgtatagcgagaacggc
CRISPR spacer 9	tttctgtctagcagaacaactatttctgaa
CRISPR spacer 10	tttgctactatgatagaaagaacattagtg
CRISPR spacer 11	tttctgtctagcagaacaactatttccgaa
CRISPR spacer 12	tttctgtctagcagaacaactatttcggcg
CRISPR spacer 13	cagaaagggttggaaggtgattcgatgaag
CRISPR spacer 14	ccaaattcatgttttaagaacccgctgata
CRISPR spacer 15	atcatttaacgtcttactgatccgcgtatt
CRISPR spacer 16	tggtggtgatatgcctaataacgaattaaa

Table S3. Structure of dsDNA templates used as a library to determine AnoCas9 PAM specificity. The PAM motif is highlighted in green.

Name	Structure (5′-3′)
Spacer-PAM _1	CAAGAAGGAGATGTCTGGGGATGTCAGGATGCATTTGTGGCCATTGTTCAAAGTG<br>TCAAGAACAAGCCTCTCTTCTTTGCCGACAAACTTTACAAATCCATGAAGGGTGCT<br>GGCACAGATGAGAA
Spacer-PAM _2	CAAGAAGGAGATGTCTGGGGATGTCAGGATGCATTTGTGGCCATTGTTCCAAGTG<br>TCAAGAACAAGCCTCTCTTCTTTGCCGACAAACTTTACAAATCCATGAAGGGTGCT<br>GGCACAGATGAGAA
Spacer-PAM _3	CAAGAAGGAGATGTCTGGGGATGTCAGGATGCATTTGTGGCCATTGTTCGAAGTG<br>TCAAGAACAAGCCTCTCTTCTTTGCCGACAAACTTTACAAATCCATGAAGGGTGCT<br>GGCACAGATGAGAA
Spacer-PAM _4	CAAGAAGGAGATGTCTGGGGATGTCAGGATGCATTTGTGGCCATTGTTCTAAGTG<br>TCAAGAACAAGCCTCTCTTCTTTGCCGACAAACTTTACAAATCCATGAAGGGTGCT<br>GGCACAGATGAGAA
Spacer-PAM _5	CAAGAAGGAGATGTCTGGGGATGTCAGGATGCATTTGTGGCCATTGATCAAAGTG<br>TCAAGAACAAGCCTCTCTTCTTTGCCGACAAACTTTACAAATCCATGAAGGGTGCT<br>GGCACAGATGAGAA
Spacer-PAM _6	CAAGAAGGAGATGTCTGGGGATGTCAGGATGCATTTGTGGCCATTGATCCAAGTG<br>TCAAGAACAAGCCTCTCTTCTTTGCCGACAAACTTTACAAATCCATGAAGGGTGCT<br>GGCACAGATGAGAA
Spacer-PAM _7	CAAGAAGGAGATGTCTGGGGATGTCAGGATGCATTTGTGGCCATTGATCGAAGTG<br>TCAAGAACAAGCCTCTCTTCTTTGCCGACAAACTTTACAAATCCATGAAGGGTGCT<br>GGCACAGATGAGAA
Spacer-PAM _8	CAAGAAGGAGATGTCTGGGGATGTCAGGATGCATTTGTGGCCATTGATCTAAGTG<br>TCAAGAACAAGCCTCTCTTCTTTGCCGACAAACTTTACAAATCCATGAAGGGTGCT<br>GGCACAGATGAGAA
Spacer-PAM _9	CAAGAAGGAGATGTCTGGGGATGTCAGGATGCATTTGTGGCCATTGCTCAAAGTG<br>TCAAGAACAAGCCTCTCTTCTTTGCCGACAAACTTTACAAATCCATGAAGGGTGCT<br>GGCACAGATGAGAA
Spacer-PAM _10	CAAGAAGGAGATGTCTGGGGATGTCAGGATGCATTTGTGGCCATTGCTCCAAGTG<br>TCAAGAACAAGCCTCTCTTCTTTGCCGACAAACTTTACAAATCCATGAAGGGTGCT<br>GGCACAGATGAGAA
Spacer-PAM _11	CAAGAAGGAGATGTCTGGGGATGTCAGGATGCATTTGTGGCCATTGCTCGAAGTG<br>TCAAGAACAAGCCTCTCTTCTTTGCCGACAAACTTTACAAATCCATGAAGGGTGCT<br>GGCACAGATGAGAA
Spacer-PAM _12	CAAGAAGGAGATGTCTGGGGATGTCAGGATGCATTTGTGGCCATTGCTCTAAGTG<br>TCAAGAACAAGCCTCTCTTCTTTGCCGACAAACTTTACAAATCCATGAAGGGTGCT<br>GGCACAGATGAGAA
Spacer-PAM _13	CAAGAAGGAGATGTCTGGGGATGTCAGGATGCATTTGTGGCCATTGGTCAAAGTG<br>TCAAGAACAAGCCTCTCTTCTTTGCCGACAAACTTTACAAATCCATGAAGGGTGCT<br>GGCACAGATGAGAA
Spacer-PAM _14	CAAGAAGGAGATGTCTGGGGATGTCAGGATGCATTTGTGGCCATTGGTCCAAGTG<br>TCAAGAACAAGCCTCTCTTCTTTGCCGACAAACTTTACAAATCCATGAAGGGTGCT<br>GGCACAGATGAGAA
Spacer-PAM _15	CAAGAAGGAGATGTCTGGGGATGTCAGGATGCATTTGTGGCCATTGGTCGAAGTG<br>TCAAGAACAAGCCTCTCTTCTTTGCCGACAAACTTTACAAATCCATGAAGGGTGCT<br>GGCACAGATGAGAA
Spacer-PAM _16	CAAGAAGGAGATGTCTGGGGATGTCAGGATGCATTTGTGGCCATTGGTCTAAGTG<br>TCAAGAACAAGCCTCTCTTCTTTGCCGACAAACTTTACAAATCCATGAAGGGTGCT<br>GGCACAGATGAGAA
Spacer-PAM _18	CAAGAAGGAGATGTCTGGGGATGTCAGGATGCATTTGTGGCCATTGGTCGACGTG<br>TCAAGAACAAGCCTCTCTTCTTTGCCGACAAACTTTACAAATCCATGAAGGGTGCT<br>GGCACAGATGAGAA

Spacer-PAM _19	CAAGAAGGAGATGTCTGGGGATGTCAGGGATGCATTTGTGGCCATTGGTCGAGGTGTCAAGAACAAGCCTCTCTTCTTTGCCGACAAACTTTACAAATCCATGAAGGGTGCTGGCACAGATGAGAA
Spacer-PAM _20	CAAGAAGGAGATGTCTGGGGATGTCAGGGATGCATTTGTGGCCATTGGTCGATGTGTCAAGAACAAGCCTCTCTTCTTTGCCGACAAACTTTACAAATCCATGAAGGGTGCTGGCACAGATGAGAA
Spacer-PAM _21	CAAGAAGGAGATGTCTGGGGATGTCAGGGATGCATTTGTGGCCATAGGTCGAAGTGTCAAGAACAAGCCTCTCTTCTTTGCCGACAAACTTTACAAATCCATGAAGGGTGCTGGCACAGATGAGAA
Spacer-PAM _22	CAAGAAGGAGATGTCTGGGGATGTCAGGGATGCATTTGTGGCCATAGGTCGACGTGTCAAGAACAAGCCTCTCTTCTTTGCCGACAAACTTTACAAATCCATGAAGGGTGCTGGCACAGATGAGAA
Spacer-PAM _23	CAAGAAGGAGATGTCTGGGGATGTCAGGGATGCATTTGTGGCCATAGGTCGAGGTGTCAAGAACAAGCCTCTCTTCTTTGCCGACAAACTTTACAAATCCATGAAGGGTGCTGGCACAGATGAGAA
Spacer-PAM _24	CAAGAAGGAGATGTCTGGGGATGTCAGGGATGCATTTGTGGCCATAGGTCGATGTGTCAAGAACAAGCCTCTCTTCTTTGCCGACAAACTTTACAAATCCATGAAGGGTGCTGGCACAGATGAGAA
Spacer-PAM _25	CAAGAAGGAGATGTCTGGGGATGTCAGGGATGCATTTGTGGCCATCGGTCGAAGTGTCAAGAACAAGCCTCTCTTCTTTGCCGACAAACTTTACAAATCCATGAAGGGTGCTGGCACAGATGAGAA
Spacer-PAM _26	CAAGAAGGAGATGTCTGGGGATGTCAGGGATGCATTTGTGGCCATCGGTCGACGTGTCAAGAACAAGCCTCTCTTCTTTGCCGACAAACTTTACAAATCCATGAAGGGTGCTGGCACAGATGAGAA
Spacer-PAM _27	CAAGAAGGAGATGTCTGGGGATGTCAGGGATGCATTTGTGGCCATCGGTCGAGGTGTCAAGAACAAGCCTCTCTTCTTTGCCGACAAACTTTACAAATCCATGAAGGGTGCTGGCACAGATGAGAA
Spacer-PAM _28	CAAGAAGGAGATGTCTGGGGATGTCAGGGATGCATTTGTGGCCATCGGTCGATGTGTCAAGAACAAGCCTCTCTTCTTTGCCGACAAACTTTACAAATCCATGAAGGGTGCTGGCACAGATGAGAA
Spacer-PAM _29	CAAGAAGGAGATGTCTGGGGATGTCAGGGATGCATTTGTGGCCATGGGTCGAAGTGTCAAGAACAAGCCTCTCTTCTTTGCCGACAAACTTTACAAATCCATGAAGGGTGCTGGCACAGATGAGAA
Spacer-PAM _30	CAAGAAGGAGATGTCTGGGGATGTCAGGGATGCATTTGTGGCCATGGGTCGACGTGTCAAGAACAAGCCTCTCTTCTTTGCCGACAAACTTTACAAATCCATGAAGGGTGCTGGCACAGATGAGAA
Spacer-PAM _31	CAAGAAGGAGATGTCTGGGGATGTCAGGGATGCATTTGTGGCCATGGGTCGAGGTGTCAAGAACAAGCCTCTCTTCTTTGCCGACAAACTTTACAAATCCATGAAGGGTGCTGGCACAGATGAGAA
Spacer-PAM _32	CAAGAAGGAGATGTCTGGGGATGTCAGGGATGCATTTGTGGCCATGGGTCGATGTGTCAAGAACAAGCCTCTCTTCTTTGCCGACAAACTTTACAAATCCATGAAGGGTGCTGGCACAGATGAGAA
Spacer-PAM-R_33	CAAGAAGGAGATGTCTGGGGATGTCAGGGATGCATTTGTGGCCATAAGACGAAGTGTCAAGAACAAGCCTCTCTTCTTTGCCGACAAACTTTACAAATCCATGAAGGGTGCTGGCACAGATGAGAA
Spacer-PAM-R_34	CAAGAAGGAGATGTCTGGGGATGTCAGGGATGCATTTGTGGCCATACGACGAAGTGTCAAGAACAAGCCTCTCTTCTTTGCCGACAAACTTTACAAATCCATGAAGGGTGCTGGCACAGATGAGAA
Spacer-PAM-R_35	CAAGAAGGAGATGTCTGGGGATGTCAGGGATGCATTTGTGGCCATAGGACGAAGTGTCAAGAACAAGCCTCTCTTCTTTGCCGACAAACTTTACAAATCCATGAAGGGTGCTGGCACAGATGAGAA
Spacer-PAM-R_36	CAAGAAGGAGATGTCTGGGGATGTCAGGGATGCATTTGTGGCCATATGACGAAGTGTCAAGAACAAGCCTCTCTTCTTTGCCGACAAACTTTACAAATCCATGAAGGGTGCTGGCACAGATGAGAA

Spacer-PAM-R_37	CAAGAAGGAGATGTCTGGGGATGTCAGGGATGCATTTGTGGCCATAAGCCGAAGTGTCAAGAACAAGCCTCTCTTCTTTGCCGACAAACTTTACAAATCCATGAAGGGTGCTGGCACAGATGAGAA
Spacer-PAM-R_38	CAAGAAGGAGATGTCTGGGGATGTCAGGGATGCATTTGTGGCCATACGCCGAAGTGTCAAGAACAAGCCTCTCTTCTTTGCCGACAAACTTTACAAATCCATGAAGGGTGCTGGCACAGATGAGAA
Spacer-PAM-R_39	CAAGAAGGAGATGTCTGGGGATGTCAGGGATGCATTTGTGGCCATAGGCCGAAGTGTCAAGAACAAGCCTCTCTTCTTTGCCGACAAACTTTACAAATCCATGAAGGGTGCTGGCACAGATGAGAA
Spacer-PAM-R_40	CAAGAAGGAGATGTCTGGGGATGTCAGGGATGCATTTGTGGCCATATGCCGAAGTGTCAAGAACAAGCCTCTCTTCTTTGCCGACAAACTTTACAAATCCATGAAGGGTGCTGGCACAGATGAGAA
Spacer-PAM-R_41	CAAGAAGGAGATGTCTGGGGATGTCAGGGATGCATTTGTGGCCATAAGGCGAAGTGTCAAGAACAAGCCTCTCTTCTTTGCCGACAAACTTTACAAATCCATGAAGGGTGCTGGCACAGATGAGAA
Spacer-PAM-R_42	CAAGAAGGAGATGTCTGGGGATGTCAGGGATGCATTTGTGGCCATACGGCGAAGTGTCAAGAACAAGCCTCTCTTCTTTGCCGACAAACTTTACAAATCCATGAAGGGTGCTGGCACAGATGAGAA
Spacer-PAM-R_43	CAAGAAGGAGATGTCTGGGGATGTCAGGGATGCATTTGTGGCCATAGGGCGAAGTGTCAAGAACAAGCCTCTCTTCTTTGCCGACAAACTTTACAAATCCATGAAGGGTGCTGGCACAGATGAGAA
Spacer-PAM-R_44	CAAGAAGGAGATGTCTGGGGATGTCAGGGATGCATTTGTGGCCATATGGCGAAGTGTCAAGAACAAGCCTCTCTTCTTTGCCGACAAACTTTACAAATCCATGAAGGGTGCTGGCACAGATGAGAA
Spacer-PAM-R_45	CAAGAAGGAGATGTCTGGGGATGTCAGGGATGCATTTGTGGCCATAAGTCGAAGTGTCAAGAACAAGCCTCTCTTCTTTGCCGACAAACTTTACAAATCCATGAAGGGTGCTGGCACAGATGAGAA
Spacer-PAM-R_46	CAAGAAGGAGATGTCTGGGGATGTCAGGGATGCATTTGTGGCCATACGTCGAAGTGTCAAGAACAAGCCTCTCTTCTTTGCCGACAAACTTTACAAATCCATGAAGGGTGCTGGCACAGATGAGAA
Spacer-PAM-R_48	CAAGAAGGAGATGTCTGGGGATGTCAGGGATGCATTTGTGGCCATATGTCGAAGTGTCAAGAACAAGCCTCTCTTCTTTGCCGACAAACTTTACAAATCCATGAAGGGTGCTGGCACAGATGAGAA
