# Supplementary material for: Expression and Functional Analysis of the Compact Thermophilic Anoxybacillus flavithermus Cas9 Nuclease
Source: Int J Mol Sci. 2023 Dec 4;24(23):17121. doi: 10.3390/ijms242317121 (PMC10707453; doi:10.3390/ijms242317121)
Supplement: Supplementary file 1 [file ijms-24-17121-s001.zip › Supplementary/Supplementary Tables.pdf]

Table S1. Amplification primers used for the generation of FAM-labeled dsDNA PAM-templates.

Name	Structure (5′-3′)
Uni-Spacer-F	caagaaggagatgtctggggatgtcagggatgcatttgtggccat
FAM-Uni-R	ttctcatctgtgccagcacccttcatggatttgtaaagtttgtcggcaaagaagaga
Spacer-PAM-R_1	ttgtcggcaaagaagagaggcttgttcttgacactttgaacaatggccacaaatgcatc
Spacer-PAM-R_2	ttgtcggcaaagaagagaggcttgttcttgacacttggaacaatggccacaaatgcatc
Spacer-PAM-R_3	ttgtcggcaaagaagagaggcttgttcttgacacttcgaacaatggccacaaatgcatc
Spacer-PAM-R_4	ttgtcggcaaagaagagaggcttgttcttgacacttagaacaatggccacaaatgcatc
Spacer-PAM-R_5	ttgtcggcaaagaagagaggcttgttcttgacactttgatcaatggccacaaatgcatc
Spacer-PAM-R_6	ttgtcggcaaagaagagaggcttgttcttgacacttggatcaatggccacaaatgcatc
Spacer-PAM-R_7	ttgtcggcaaagaagagaggcttgttcttgacacttcgatcaatggccacaaatgcatc
Spacer-PAM-R_8	ttgtcggcaaagaagagaggcttgttcttgacacttagatcaatggccacaaatgcatc
Spacer-PAM-R_9	ttgtcggcaaagaagagaggcttgttcttgacactttgagcaatggccacaaatgcatc
Spacer-PAM-R_10	ttgtcggcaaagaagagaggcttgttcttgacacttggagcaatggccacaaatgcatc
Spacer-PAM-R_11	ttgtcggcaaagaagagaggcttgttcttgacacttcgagcaatggccacaaatgcatc
Spacer-PAM-R_12	ttgtcggcaaagaagagaggcttgttcttgacacttagagcaatggccacaaatgcatc
Spacer-PAM-R_13	ttgtcggcaaagaagagaggcttgttcttgacactttgaccaatggccacaaatgcatc
Spacer-PAM-R_14	ttgtcggcaaagaagagaggcttgttcttgacacttggaccaatggccacaaatgcatc
Spacer-PAM-R_15	ttgtcggcaaagaagagaggcttgttcttgacacttcgaccaatggccacaaatgcatc
Spacer-PAM-R_16	ttgtcggcaaagaagagaggcttgttcttgacacttagaccaatggccacaaatgcatc
Spacer-PAM-R_18	ttgtcggcaaagaagagaggcttgttcttgacacgtcgaccaatggccacaaatgcatc
Spacer-PAM-R_19	ttgtcggcaaagaagagaggcttgttcttgacacctcgaccaatggccacaaatgcatc
Spacer-PAM-R_20	ttgtcggcaaagaagagaggcttgttcttgacacatcgaccaatggccacaaatgcatc
Spacer-PAM-R_21	ttgtcggcaaagaagagaggcttgttcttgacacttcgacctatggccacaaatgcatc
Spacer-PAM-R_22	ttgtcggcaaagaagagaggcttgttcttgacacgtcgacctatggccacaaatgcatc
Spacer-PAM-R_23	ttgtcggcaaagaagagaggcttgttcttgacacctcgacctatggccacaaatgcatc
Spacer-PAM-R_24	ttgtcggcaaagaagagaggcttgttcttgacacatcgacctatggccacaaatgcatc
Spacer-PAM-R_25	ttgtcggcaaagaagagaggcttgttcttgacacttcgaccgatggccacaaatgcatc
Spacer-PAM-R_26	ttgtcggcaaagaagagaggcttgttcttgacacgtcgaccgatggccacaaatgcatc
Spacer-PAM-R_27	ttgtcggcaaagaagagaggcttgttcttgacacctcgaccgatggccacaaatgcatc
Spacer-PAM-R_28	ttgtcggcaaagaagagaggcttgttcttgacacatcgaccgatggccacaaatgcatc
Spacer-PAM-R_29	ttgtcggcaaagaagagaggcttgttcttgacacttcgacccatggccacaaatgcatc
Spacer-PAM-R_30	ttgtcggcaaagaagagaggcttgttcttgacacgtcgacccatggccacaaatgcatc
Spacer-PAM-R_31	ttgtcggcaaagaagagaggcttgttcttgacacctcgacccatggccacaaatgcatc
Spacer-PAM-R_32	ttgtcggcaaagaagagaggcttgttcttgacacatcgacccatggccacaaatgcatc
Spacer-PAM-R_33	ttgtcggcaaagaagagaggcttgttcttgacacttcgtcttatggccacaaatgcatc
Spacer-PAM-R_34	ttgtcggcaaagaagagaggcttgttcttgacacttcgtcgtatggccacaaatgcatc
Spacer-PAM-R_35	ttgtcggcaaagaagagaggcttgttcttgacacttcgtcctatggccacaaatgcatc
Spacer-PAM-R_36	ttgtcggcaaagaagagaggcttgttcttgacacttcgtcatatggccacaaatgcatc
Spacer-PAM-R_37	ttgtcggcaaagaagagaggcttgttcttgacacttcggcttatggccacaaatgcatc
Spacer-PAM-R_38	ttgtcggcaaagaagagaggcttgttcttgacacttcggcgtatggccacaaatgcatc
Spacer-PAM-R_39	ttgtcggcaaagaagagaggcttgttcttgacacttcggcctatggccacaaatgcatc
Spacer-PAM-R_40	ttgtcggcaaagaagagaggcttgttcttgacacttcggcatatggccacaaatgcatc
Spacer-PAM-R_41	ttgtcggcaaagaagagaggcttgttcttgacacttcgccttatggccacaaatgcatc
Spacer-PAM-R_42	ttgtcggcaaagaagagaggcttgttcttgacacttcgccgtatggccacaaatgcatc
Spacer-PAM-R_43	ttgtcggcaaagaagagaggcttgttcttgacacttcgccctatggccacaaatgcatc
Spacer-PAM-R_44	ttgtcggcaaagaagagaggcttgttcttgacacttcgccatatggccacaaatgcatc
Spacer-PAM-R_45	ttgtcggcaaagaagagaggcttgttcttgacacttcgacttatggccacaaatgcatc
Spacer-PAM-R_46	ttgtcggcaaagaagagaggcttgttcttgacacttcgacgtatggccacaaatgcatc
Spacer-PAM-R_48	ttgtcggcaaagaagagaggcttgttcttgacacttcgacatatggccacaaatgcatc
Spacer-PAM-R_33	ttgtcggcaaagaagagaggcttgttcttgacacttcgtcttatggccacaaatgcatc

Table S2. Sequences of CRISPR array elements derived from the *Anoxybacillus flavithermus* genome.

Name	Structure (5′-3′)
CRISPR repeat	gtaatagttcccctgaggttattgctgtgttatgat
CRISPR spacer 1	aaaatgacacacttatttgataaaaagaaa
CRISPR spacer 2	ccgaaagttttagactcactataaatcctc
CRISPR spacer 3	aaaaaggagatcatgaaaaaagcagtagct
CRISPR spacer 4	cgtgggtcaattcggtaaatggaagttttt
CRISPR spacer 5	tgctacacttttgcaccgaaacggagcgga
CRISPR spacer 6	agaccagtccaactagaaattaattttaga
CRISPR spacer 7	tgaagaaaaagaaaactgtagagaaacaac
CRISPR spacer 8	tcatcagcaaaaatgtatagcgagaacggc
CRISPR spacer 9	tttctgtctagcagaacaactatttctgaa
CRISPR spacer 10	tttgctactatgatagaaagaacattagtg
CRISPR spacer 11	tttctgtctagcagaacaactatttccgaa
CRISPR spacer 12	tttctgtctagcagaacaactatttcggcg
CRISPR spacer 13	cagaaagggttggaaggtgattcgatgaag
CRISPR spacer 14	ccaaattcatgttttaagaacccgctgata
CRISPR spacer 15	atcatttaacgtcttactgatccgcgtatt
CRISPR spacer 16	tggtggtgatatgcctaataacgaattaaa

Table S3. Structure of dsDNA templates used as a library to determine AnoCas9 PAM specificity. The PAM motif is highlighted in green.

Name	Structure (5′-3′)
Spacer-PAM _1	CAAGAAGGAGATGTCTGGGGATGTCAGGGATGCATTTGTGGCCATTGTTCAAAGTG<br>TCAAGAACAAGCCTCTCTTCTTTGCCGACAAACTTTACAAATCCATGAAGGGTGCT<br>GGCACAGATGAGAA
Spacer-PAM _2	CAAGAAGGAGATGTCTGGGGATGTCAGGGATGCATTTGTGGCCATTGTTCCAAGTG<br>TCAAGAACAAGCCTCTCTTCTTTGCCGACAAACTTTACAAATCCATGAAGGGTGCT<br>GGCACAGATGAGAA
Spacer-PAM _3	CAAGAAGGAGATGTCTGGGGATGTCAGGGATGCATTTGTGGCCATTGTTCGAAGTG<br>TCAAGAACAAGCCTCTCTTCTTTGCCGACAAACTTTACAAATCCATGAAGGGTGCT<br>GGCACAGATGAGAA
Spacer-PAM _4	CAAGAAGGAGATGTCTGGGGATGTCAGGGATGCATTTGTGGCCATTGTTCTAAGTG<br>TCAAGAACAAGCCTCTCTTCTTTGCCGACAAACTTTACAAATCCATGAAGGGTGCT<br>GGCACAGATGAGAA
Spacer-PAM _5	CAAGAAGGAGATGTCTGGGGATGTCAGGGATGCATTTGTGGCCATTGATCAAAGTG<br>TCAAGAACAAGCCTCTCTTCTTTGCCGACAAACTTTACAAATCCATGAAGGGTGCT<br>GGCACAGATGAGAA
Spacer-PAM _6	CAAGAAGGAGATGTCTGGGGATGTCAGGGATGCATTTGTGGCCATTGATCCAAGTG<br>TCAAGAACAAGCCTCTCTTCTTTGCCGACAAACTTTACAAATCCATGAAGGGTGCT<br>GGCACAGATGAGAA
Spacer-PAM _7	CAAGAAGGAGATGTCTGGGGATGTCAGGGATGCATTTGTGGCCATTGATCGAAGTG<br>TCAAGAACAAGCCTCTCTTCTTTGCCGACAAACTTTACAAATCCATGAAGGGTGCT<br>GGCACAGATGAGAA
Spacer-PAM _8	CAAGAAGGAGATGTCTGGGGATGTCAGGGATGCATTTGTGGCCATTGATCTAAGTG<br>TCAAGAACAAGCCTCTCTTCTTTGCCGACAAACTTTACAAATCCATGAAGGGTGCT<br>GGCACAGATGAGAA
Spacer-PAM _9	CAAGAAGGAGATGTCTGGGGATGTCAGGGATGCATTTGTGGCCATTGCTCAAAGTG<br>TCAAGAACAAGCCTCTCTTCTTTGCCGACAAACTTTACAAATCCATGAAGGGTGCT<br>GGCACAGATGAGAA
Spacer-PAM _10	CAAGAAGGAGATGTCTGGGGATGTCAGGGATGCATTTGTGGCCATTGCTCCAAGTG<br>TCAAGAACAAGCCTCTCTTCTTTGCCGACAAACTTTACAAATCCATGAAGGGTGCT<br>GGCACAGATGAGAA
Spacer-PAM _11	CAAGAAGGAGATGTCTGGGGATGTCAGGGATGCATTTGTGGCCATTGCTCGAAGTG<br>TCAAGAACAAGCCTCTCTTCTTTGCCGACAAACTTTACAAATCCATGAAGGGTGCT<br>GGCACAGATGAGAA
Spacer-PAM _12	CAAGAAGGAGATGTCTGGGGATGTCAGGGATGCATTTGTGGCCATTGCTCTAAGTG<br>TCAAGAACAAGCCTCTCTTCTTTGCCGACAAACTTTACAAATCCATGAAGGGTGCT<br>GGCACAGATGAGAA
Spacer-PAM _13	CAAGAAGGAGATGTCTGGGGATGTCAGGGATGCATTTGTGGCCATTGGTCAAAGTG<br>TCAAGAACAAGCCTCTCTTCTTTGCCGACAAACTTTACAAATCCATGAAGGGTGCT<br>GGCACAGATGAGAA
Spacer-PAM _14	CAAGAAGGAGATGTCTGGGGATGTCAGGGATGCATTTGTGGCCATTGGTCCAAGTG<br>TCAAGAACAAGCCTCTCTTCTTTGCCGACAAACTTTACAAATCCATGAAGGGTGCT<br>GGCACAGATGAGAA
Spacer-PAM _15	CAAGAAGGAGATGTCTGGGGATGTCAGGGATGCATTTGTGGCCATTGGTCGAAGTG<br>TCAAGAACAAGCCTCTCTTCTTTGCCGACAAACTTTACAAATCCATGAAGGGTGCT<br>GGCACAGATGAGAA
Spacer-PAM _16	CAAGAAGGAGATGTCTGGGGATGTCAGGGATGCATTTGTGGCCATTGGTCTAAGTG<br>TCAAGAACAAGCCTCTCTTCTTTGCCGACAAACTTTACAAATCCATGAAGGGTGCT<br>GGCACAGATGAGAA
Spacer-PAM _18	CAAGAAGGAGATGTCTGGGGATGTCAGGGATGCATTTGTGGCCATTGGTCGACGTG<br>TCAAGAACAAGCCTCTCTTCTTTGCCGACAAACTTTACAAATCCATGAAGGGTGCT<br>GGCACAGATGAGAA

Spacer-PAM _19	CAAGAAGGAGATGTCTGGGGATGTCAGGGATGCATTTGTGGCCATTGGTCGAGGTGTCAAGAACAAGCCTCTCTTCTTTGCCGACAAACTTTACAAATCCATGAAGGGTGCTGGCACAGATGAGAA
Spacer-PAM _20	CAAGAAGGAGATGTCTGGGGATGTCAGGGATGCATTTGTGGCCATTGGTCGATGTGTCAAGAACAAGCCTCTCTTCTTTGCCGACAAACTTTACAAATCCATGAAGGGTGCTGGCACAGATGAGAA
Spacer-PAM _21	CAAGAAGGAGATGTCTGGGGATGTCAGGGATGCATTTGTGGCCATAGGTCGAAGTGTCAAGAACAAGCCTCTCTTCTTTGCCGACAAACTTTACAAATCCATGAAGGGTGCTGGCACAGATGAGAA
Spacer-PAM _22	CAAGAAGGAGATGTCTGGGGATGTCAGGGATGCATTTGTGGCCATAGGTCGACGTGTCAAGAACAAGCCTCTCTTCTTTGCCGACAAACTTTACAAATCCATGAAGGGTGCTGGCACAGATGAGAA
Spacer-PAM _23	CAAGAAGGAGATGTCTGGGGATGTCAGGGATGCATTTGTGGCCATAGGTCGAGGTGTCAAGAACAAGCCTCTCTTCTTTGCCGACAAACTTTACAAATCCATGAAGGGTGCTGGCACAGATGAGAA
Spacer-PAM _24	CAAGAAGGAGATGTCTGGGGATGTCAGGGATGCATTTGTGGCCATAGGTCGATGTGTCAAGAACAAGCCTCTCTTCTTTGCCGACAAACTTTACAAATCCATGAAGGGTGCTGGCACAGATGAGAA
Spacer-PAM _25	CAAGAAGGAGATGTCTGGGGATGTCAGGGATGCATTTGTGGCCATCGGTCGAAGTGTCAAGAACAAGCCTCTCTTCTTTGCCGACAAACTTTACAAATCCATGAAGGGTGCTGGCACAGATGAGAA
Spacer-PAM _26	CAAGAAGGAGATGTCTGGGGATGTCAGGGATGCATTTGTGGCCATCGGTCGACGTGTCAAGAACAAGCCTCTCTTCTTTGCCGACAAACTTTACAAATCCATGAAGGGTGCTGGCACAGATGAGAA
Spacer-PAM _27	CAAGAAGGAGATGTCTGGGGATGTCAGGGATGCATTTGTGGCCATCGGTCGAGGTGTCAAGAACAAGCCTCTCTTCTTTGCCGACAAACTTTACAAATCCATGAAGGGTGCTGGCACAGATGAGAA
Spacer-PAM _28	CAAGAAGGAGATGTCTGGGGATGTCAGGGATGCATTTGTGGCCATCGGTCGATGTGTCAAGAACAAGCCTCTCTTCTTTGCCGACAAACTTTACAAATCCATGAAGGGTGCTGGCACAGATGAGAA
Spacer-PAM _29	CAAGAAGGAGATGTCTGGGGATGTCAGGGATGCATTTGTGGCCATGGGTCGAAGTGTCAAGAACAAGCCTCTCTTCTTTGCCGACAAACTTTACAAATCCATGAAGGGTGCTGGCACAGATGAGAA
Spacer-PAM _30	CAAGAAGGAGATGTCTGGGGATGTCAGGGATGCATTTGTGGCCATGGGTCGACGTGTCAAGAACAAGCCTCTCTTCTTTGCCGACAAACTTTACAAATCCATGAAGGGTGCTGGCACAGATGAGAA
Spacer-PAM _31	CAAGAAGGAGATGTCTGGGGATGTCAGGGATGCATTTGTGGCCATGGGTCGAGGTGTCAAGAACAAGCCTCTCTTCTTTGCCGACAAACTTTACAAATCCATGAAGGGTGCTGGCACAGATGAGAA
Spacer-PAM _32	CAAGAAGGAGATGTCTGGGGATGTCAGGGATGCATTTGTGGCCATGGGTCGATGTGTCAAGAACAAGCCTCTCTTCTTTGCCGACAAACTTTACAAATCCATGAAGGGTGCTGGCACAGATGAGAA
Spacer-PAM-R_33	CAAGAAGGAGATGTCTGGGGATGTCAGGGATGCATTTGTGGCCATAAGACGAAGTGTCAAGAACAAGCCTCTCTTCTTTGCCGACAAACTTTACAAATCCATGAAGGGTGCTGGCACAGATGAGAA
Spacer-PAM-R_34	CAAGAAGGAGATGTCTGGGGATGTCAGGGATGCATTTGTGGCCATACGACGAAGTGTCAAGAACAAGCCTCTCTTCTTTGCCGACAAACTTTACAAATCCATGAAGGGTGCTGGCACAGATGAGAA
Spacer-PAM-R_35	CAAGAAGGAGATGTCTGGGGATGTCAGGGATGCATTTGTGGCCATAGGACGAAGTGTCAAGAACAAGCCTCTCTTCTTTGCCGACAAACTTTACAAATCCATGAAGGGTGCTGGCACAGATGAGAA
Spacer-PAM-R_36	CAAGAAGGAGATGTCTGGGGATGTCAGGGATGCATTTGTGGCCATATGACGAAGTGTCAAGAACAAGCCTCTCTTCTTTGCCGACAAACTTTACAAATCCATGAAGGGTGCTGGCACAGATGAGAA

Spacer-PAM-R_37	CAAGAAGGAGATGTCTGGGGATGTCAGGGATGCATTTGTGGCCATAAGCCGAAGTGTCAAGAACAAGCCTCTCTTCTTTGCCGACAAACTTTACAAATCCATGAAGGGTGCTGGCACAGATGAGAA
Spacer-PAM-R_38	CAAGAAGGAGATGTCTGGGGATGTCAGGGATGCATTTGTGGCCATACGCCGAAGTGTCAAGAACAAGCCTCTCTTCTTTGCCGACAAACTTTACAAATCCATGAAGGGTGCTGGCACAGATGAGAA
Spacer-PAM-R_39	CAAGAAGGAGATGTCTGGGGATGTCAGGGATGCATTTGTGGCCATAGGCCGAAGTGTCAAGAACAAGCCTCTCTTCTTTGCCGACAAACTTTACAAATCCATGAAGGGTGCTGGCACAGATGAGAA
Spacer-PAM-R_40	CAAGAAGGAGATGTCTGGGGATGTCAGGGATGCATTTGTGGCCATATGCCGAAGTGTCAAGAACAAGCCTCTCTTCTTTGCCGACAAACTTTACAAATCCATGAAGGGTGCTGGCACAGATGAGAA
Spacer-PAM-R_41	CAAGAAGGAGATGTCTGGGGATGTCAGGGATGCATTTGTGGCCATAAGGCGAAGTGTCAAGAACAAGCCTCTCTTCTTTGCCGACAAACTTTACAAATCCATGAAGGGTGCTGGCACAGATGAGAA
Spacer-PAM-R_42	CAAGAAGGAGATGTCTGGGGATGTCAGGGATGCATTTGTGGCCATACGGCGAAGTGTCAAGAACAAGCCTCTCTTCTTTGCCGACAAACTTTACAAATCCATGAAGGGTGCTGGCACAGATGAGAA
Spacer-PAM-R_43	CAAGAAGGAGATGTCTGGGGATGTCAGGGATGCATTTGTGGCCATAGGGCGAAGTGTCAAGAACAAGCCTCTCTTCTTTGCCGACAAACTTTACAAATCCATGAAGGGTGCTGGCACAGATGAGAA
Spacer-PAM-R_44	CAAGAAGGAGATGTCTGGGGATGTCAGGGATGCATTTGTGGCCATATGGCGAAGTGTCAAGAACAAGCCTCTCTTCTTTGCCGACAAACTTTACAAATCCATGAAGGGTGCTGGCACAGATGAGAA
Spacer-PAM-R_45	CAAGAAGGAGATGTCTGGGGATGTCAGGGATGCATTTGTGGCCATAAGTCGAAGTGTCAAGAACAAGCCTCTCTTCTTTGCCGACAAACTTTACAAATCCATGAAGGGTGCTGGCACAGATGAGAA
Spacer-PAM-R_46	CAAGAAGGAGATGTCTGGGGATGTCAGGGATGCATTTGTGGCCATACGTCGAAGTGTCAAGAACAAGCCTCTCTTCTTTGCCGACAAACTTTACAAATCCATGAAGGGTGCTGGCACAGATGAGAA
Spacer-PAM-R_48	CAAGAAGGAGATGTCTGGGGATGTCAGGGATGCATTTGTGGCCATATGTCGAAGTGTCAAGAACAAGCCTCTCTTCTTTGCCGACAAACTTTACAAATCCATGAAGGGTGCTGGCACAGATGAGAA
