# Supplementary figures and images for: Expression and Functional Analysis of the Compact Thermophilic Anoxybacillus flavithermus Cas9 Nuclease
Source: Int J Mol Sci. 2023 Dec 4;24(23):17121. doi: 10.3390/ijms242317121 (PMC10707453; doi:10.3390/ijms242317121)

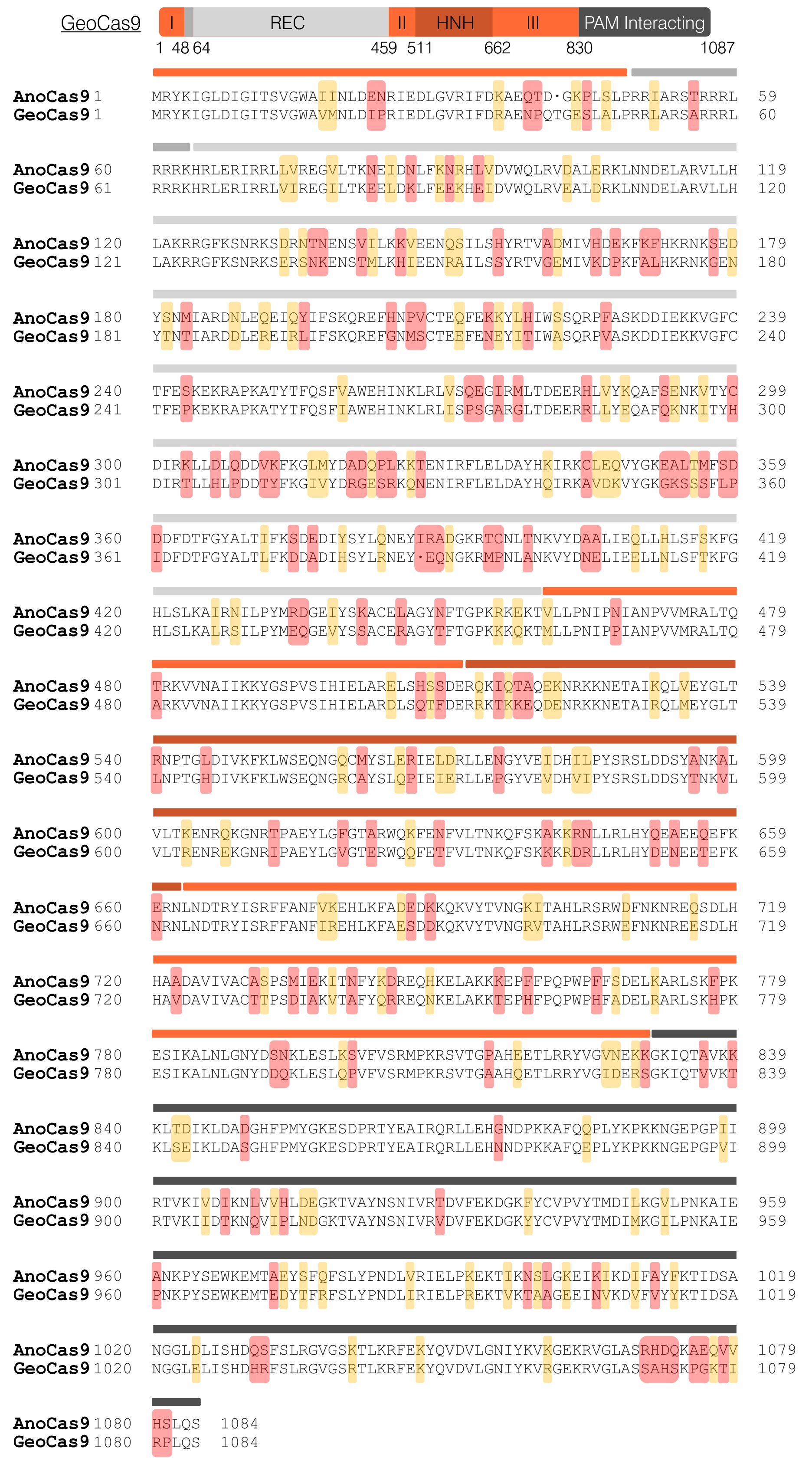

Supplement: Supplementary file 1 [file ijms-24-17121-s001.zip › Supplementary/FigureS1.jpg]

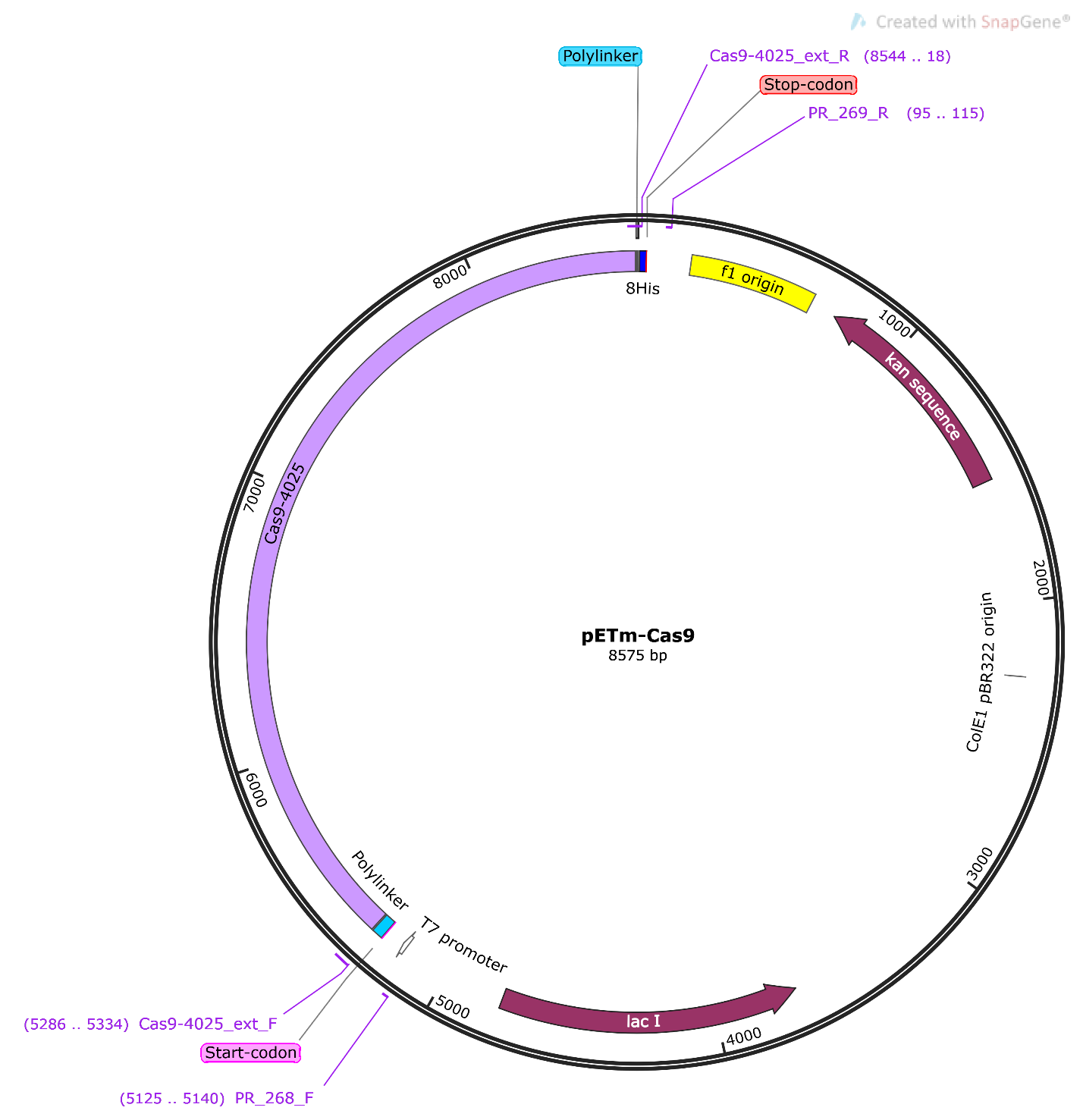

Supplement: Supplementary file 1 [file ijms-24-17121-s001.zip › Supplementary/FigureS2.png]

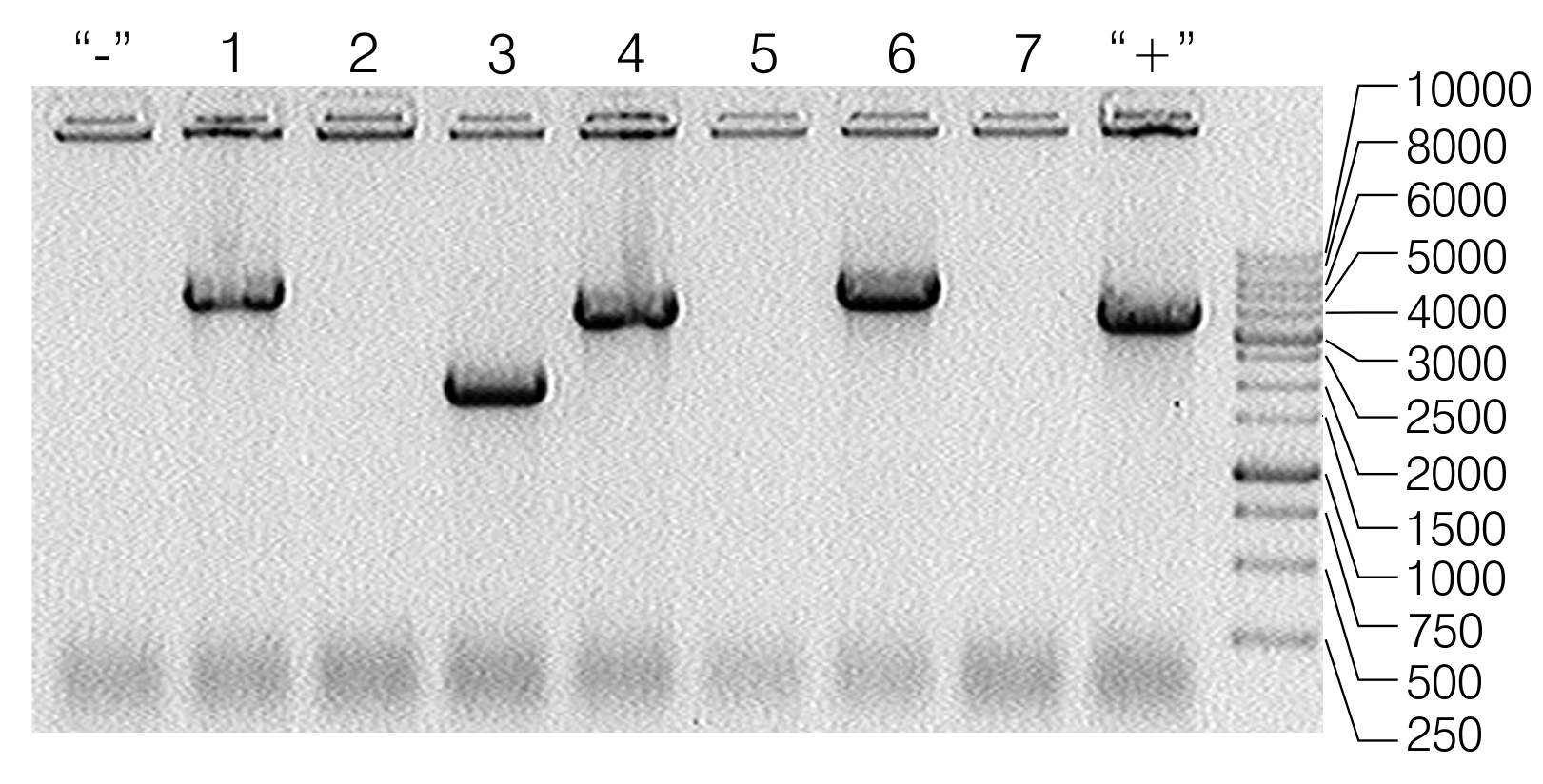

Supplement: Supplementary file 1 [file ijms-24-17121-s001.zip › Supplementary/FigureS3.png]

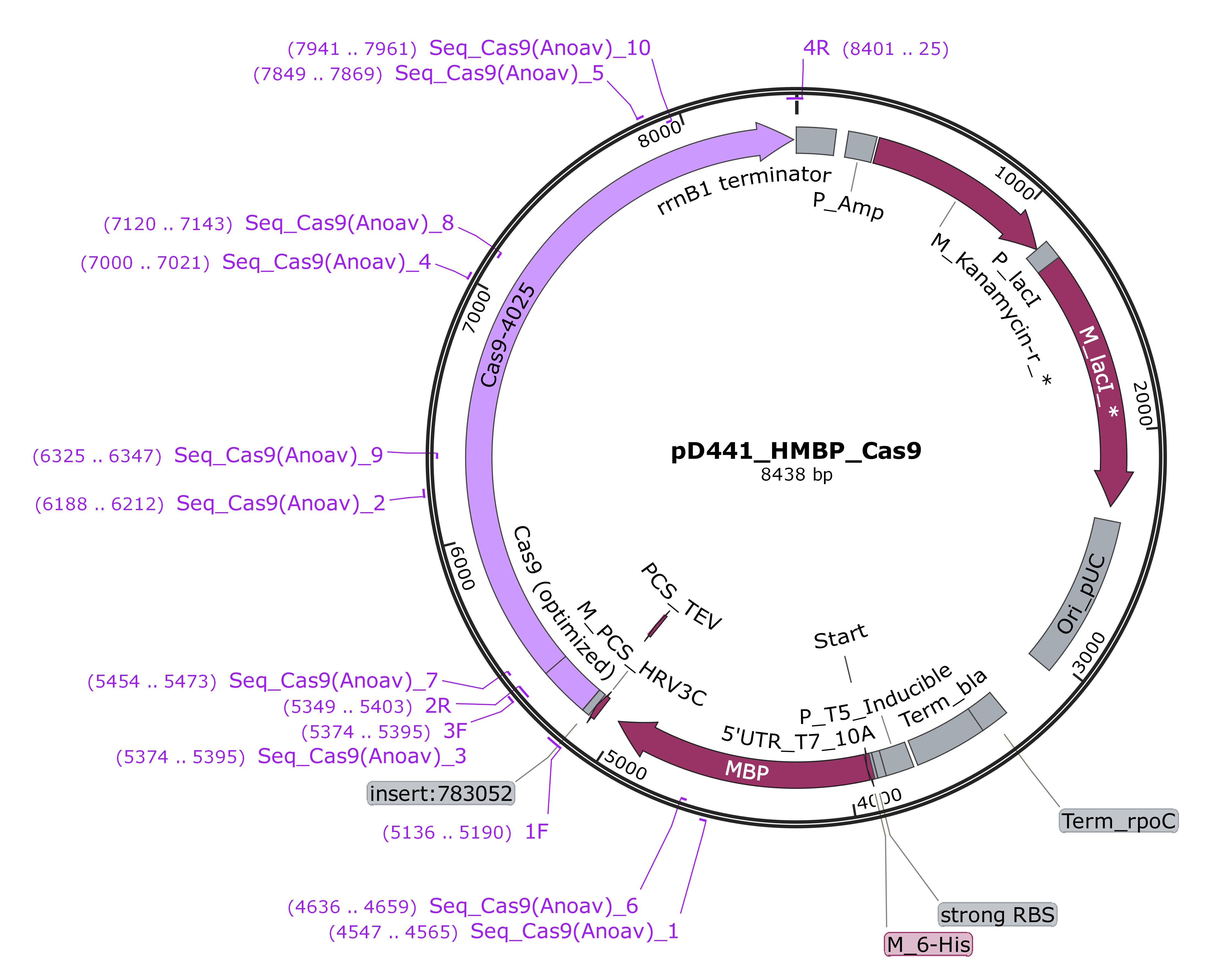

Supplement: Supplementary file 1 [file ijms-24-17121-s001.zip › Supplementary/FigureS4.png]

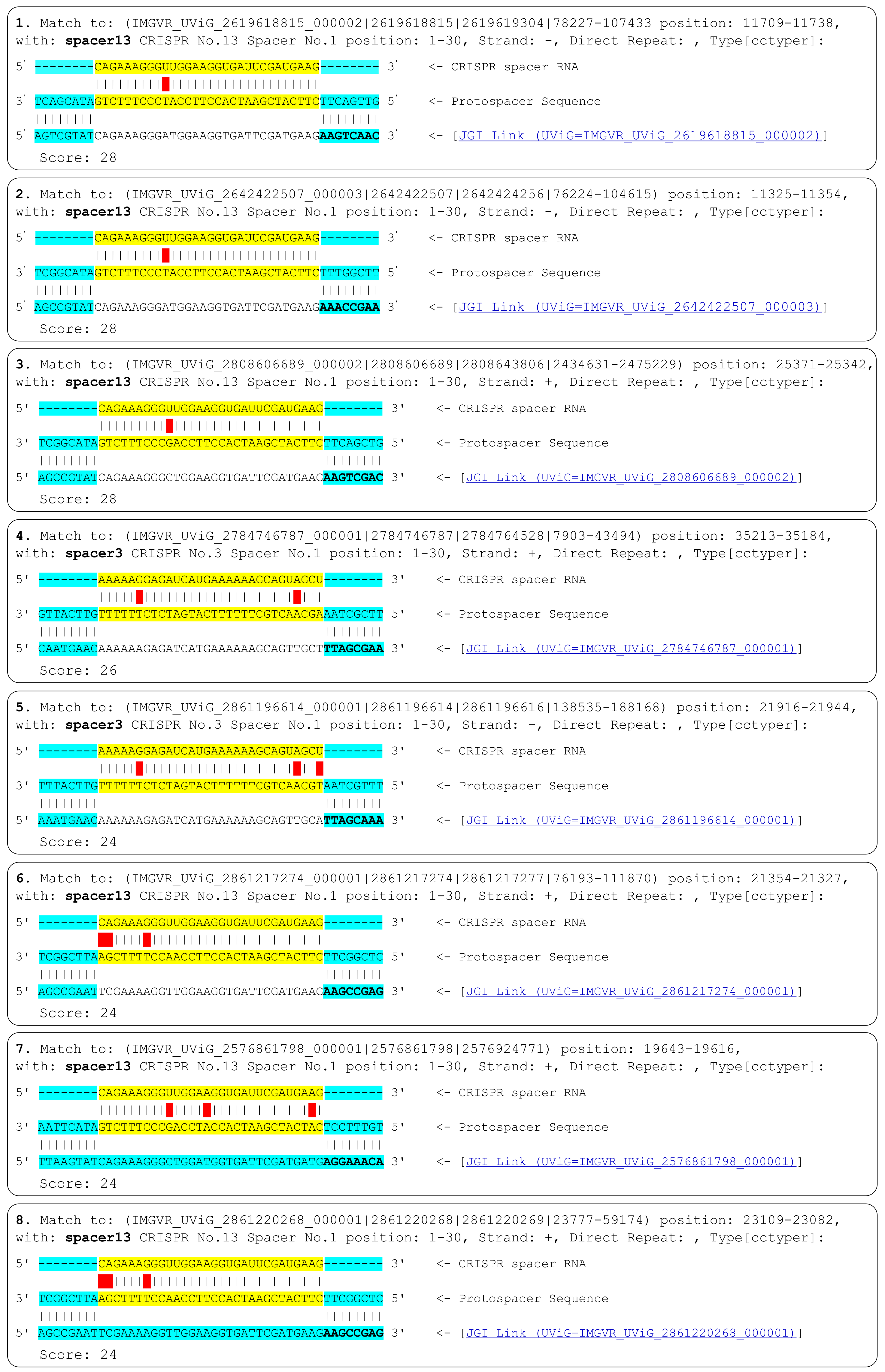

Supplement: Supplementary file 1 [file ijms-24-17121-s001.zip › Supplementary/FigureS5.png]

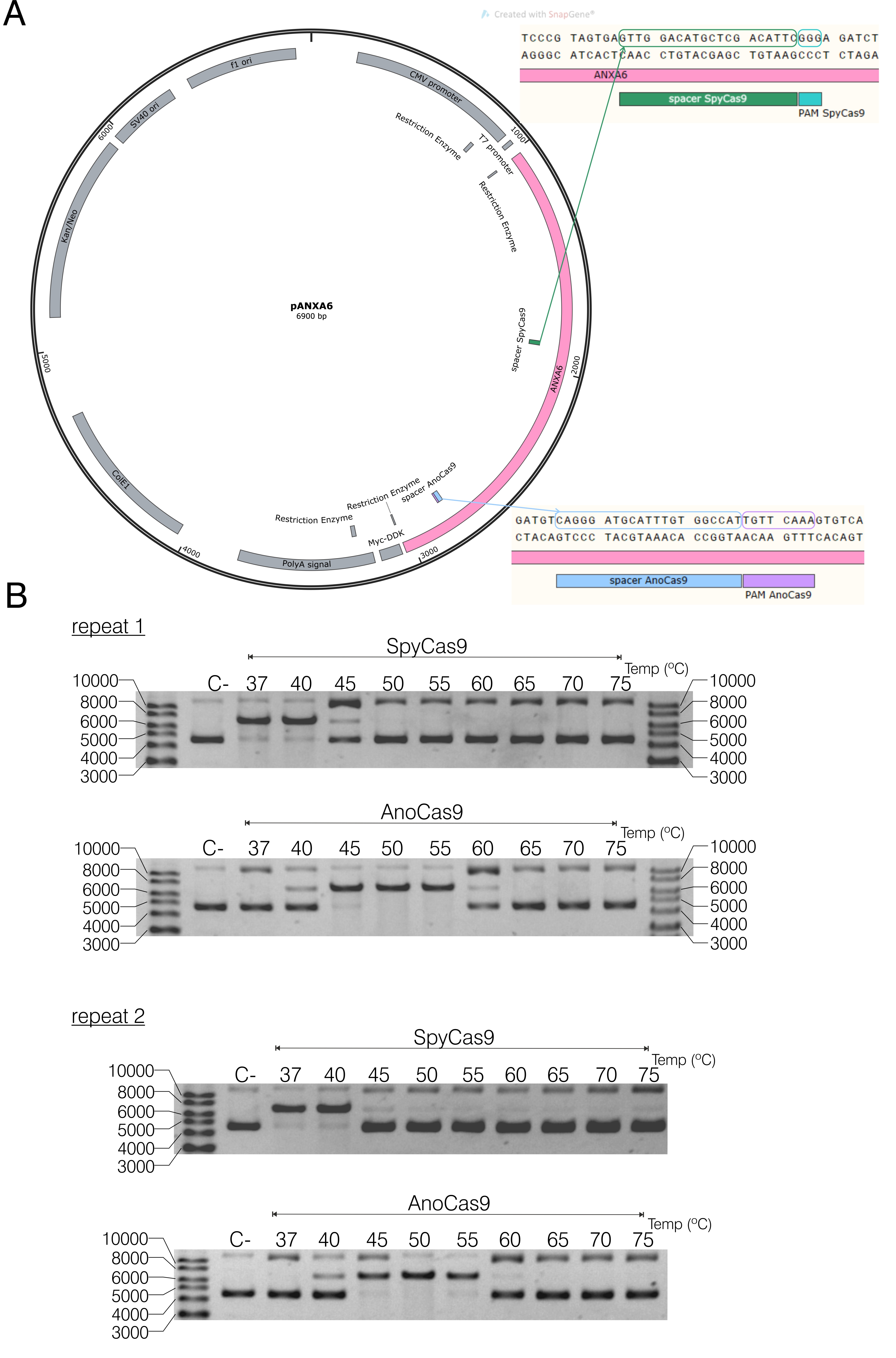

Supplement: Supplementary file 1 [file ijms-24-17121-s001.zip › Supplementary/FigureS6.jpg]

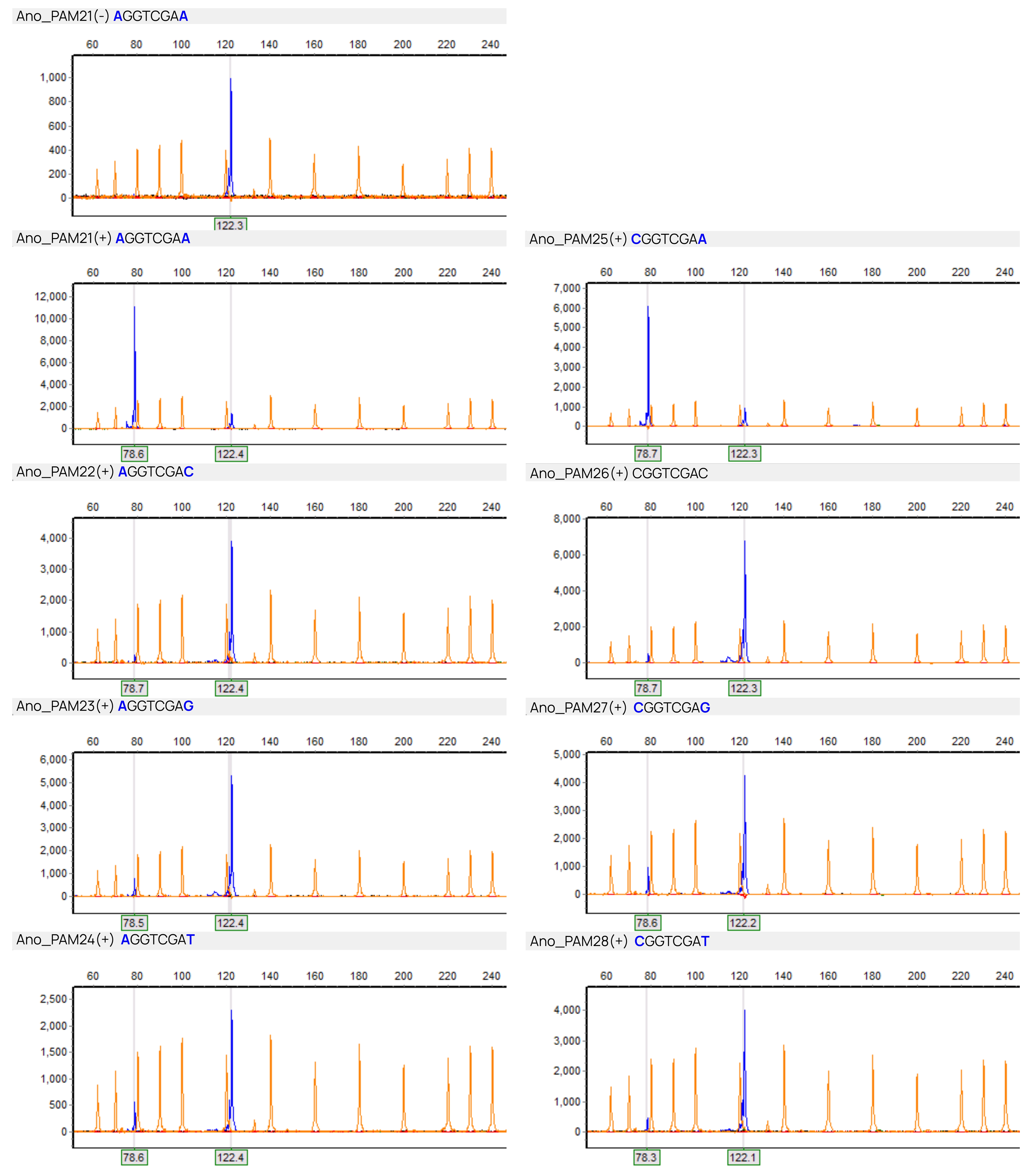

Supplement: Supplementary file 1 [file ijms-24-17121-s001.zip › Supplementary/FigureS7.jpg]
